# Idiopathic male infertility revisited: can redox endophenotypes reframe the ‘idiopathic’ label?

**DOI:** 10.3389/fendo.2026.1747453

**Published:** 2026-03-03

**Authors:** Pallav Sengupta, Sulagna Dutta, Ralf Henkel, Israel Maldonado Rosas, Shubhadeep Roychoudhury

**Affiliations:** 1 Department of Biomedical Sciences, College of Medicine, Gulf Medical University, Ajman, United Arab Emirates; 2 Basic Medical Sciences Department, College of Medicine, Ajman University, Ajman, United Arab Emirates; 3 Department of Medical Bioscience; University of the Western Cape, Belville, South Africa; 4 Citmer Reproductive Medicine, Mexico City, Mexico; 5 Department of Life Science and Bioinformatics, Assam University, Silchar, India

**Keywords:** assisted reproductive technology, male fertility, oxidative stress, redox biomarkers, reductive stress, sperm DNA damage

## Abstract

Male infertility accounts for nearly half of all infertility cases worldwide, yet up to 30-40% of affected men remain categorized as ‘idiopathic’, reflecting limitations of conventional diagnostics such as semen analysis, hormonal profiling, karyotyping, and Y-chromosome microdeletion testing. These methods describe sperm quantity and morphology but fail to uncover underlying molecular dysfunctions. Increasing evidence suggests that oxidative stress plays a central role in driving sperm damage, encompassing lipid peroxidation, protein oxidation, mitochondrial dysfunction, DNA fragmentation, and epigenetic instability. Such diverse pathways highlight the inadequacy of a single idiopathic category and necessitate mechanistic stratification. This evidence-based study proposes redox endophenotypes, including hyper- and hypo-oxidative, DNA damage-dominant, mitochondrial dysfunction, epigenetic-redox, and inflammatory-redox phenotypes, as a framework for reclassifying idiopathic male infertility. Each phenotype integrates specific biomarkers, such as oxidation-reduction potential (ORP), isoprostanes, 8-hydroxy-2′-deoxyguanosine, or advanced oxidation protein products, with distinct functional impairments and clinical outcomes. Redox endophenotyping offers diagnostic refinement, guides personalized antioxidant or adjunctive therapy, and informs clinicians and embryologists for appropriate assisted reproductive technology (ART) protocols by counselling and treating the patient, and tailoring sperm selection and preparation strategies. Future directions include multi-center validation of redox assays, integration with omics and exposome data, and application of artificial intelligence for biomarker-driven algorithms. Recognizing redox endophenotypes not only reduces the reliance on exclusionary ‘idiopathic’ diagnoses but also advances precision andrology, improving patient diagnostics and care, reproductive outcomes and mitigating intergenerational health risks.

## 1 Introduction

Male infertility represents a significant global health burden, contributing to nearly half of all infertility cases among couples (1). Despite advances in diagnostics, approximately 30-40% of infertile men are classified as having idiopathic infertility, reflecting the absence of identifiable etiological factors following standard clinical evaluations, with reported prevalence varying across populations, diagnostic criteria, and study settings (2). This persistent category underscores the limitations of conventional diagnostic modalities such as semen analysis, hormonal profiling, karyotyping, and Y chromosome microdeletion screening, which provide descriptive but not mechanistic insights into impaired male fertility (3). A growing body of evidence implicates oxidative stress (OS) as a central player in male infertility pathophysiology (4, 5). Reactive oxygen species (ROS), when exceeding physiological thresholds, can disrupt sperm membrane integrity, mitochondrial bioenergetics, and nuclear as well as epigenetic stability (6). The dichotomy between the physiological roles of ROS in capacitation and fertilization versus their pathological impact through OS highlights the necessity of assessing redox balance as a functional biomarker (7). However, current laboratory assessments remain fragmented, often lacking reproducibility and standardization, thereby failing to provide clinically actionable insights (8, 9).

Conventional semen analysis focuses on sperm concentration, motility, and morphology, yet these parameters do not capture the underlying molecular disturbances that compromise fertilization potential (10). Consequently, a substantial number of men continue to be grouped into the ‘idiopathic’ category, limiting personalized treatment strategies and affecting outcomes in assisted reproductive technologies (ART) (3, 11). While OS represents a central and unifying mechanism in male infertility, it does not act in isolation. Redox imbalance interacts with genetic, epigenetic, endocrine, immunological, and environmental factors, and limitations related to biomarker standardization, temporal variability, and causality warrant cautious interpretation and individualized clinical application. This review revisits idiopathic male infertility (IMI) through the lens of redox biology, proposing redox-based endophenotypes as a conceptual framework for stratification. In this context, a ‘redox endophenotype’ is defined as a quantifiable, intermediate biological state characterized by distinct patterns of oxidative–reductive imbalance that link molecular redox dysregulation with functional sperm impairment and clinical infertility phenotypes. By defining distinct mechanistic subgroups such as hyper-oxidative, DNA damage-dominant, mitochondrial dysfunction, epigenetic-oxidative, and inflammatory-oxidative phenotypes, we seek to move beyond descriptive diagnostics toward precision andrology. Such an approach holds the promise of refining diagnosis, guiding personalized antioxidant and adjunctive therapies, and ultimately reducing the reliance on the ambiguous label of ‘idiopathic’ infertility.

## 2 Idiopathic male infertility: a clinical and conceptual overview

### 2.1 Definitions and diagnostic challenges of idiopathic male infertility

IMI is broadly defined as the inability to conceive after at least 12 months of unprotected intercourse, in the absence of identifiable female factors, where standard diagnostic evaluations in men fail to reveal a clear etiology (2). Unlike defined conditions such as varicocele, hypogonadotropic hypogonadism, obstructive azoospermia, or genetic abnormalities, idiopathic infertility represents a residual category encompassing 30-40% of infertile men worldwide (12). The lack of definitional precision reflects not only biological heterogeneity but also diagnostic insufficiency, highlighting the limitations of conventional approaches in andrology.

Diagnostic pathway for male infertility typically includes semen analysis, hormonal profiling, scrotal ultrasonography, karyotyping, and Y-chromosome microdeletion testing (13). While these assessments can identify gross abnormalities, they lack sensitivity to subtle functional, molecular, or environmental contributors to sperm dysfunction. For example, semen analysis classifies patients into oligozoospermia, asthenozoospermia, teratozoospermia, or normozoospermia (10), yet a significant proportion of men with ‘normal’ semen parameters remain infertile, pointing to cryptic defects such as oxidative DNA damage, mitochondrial dysfunction, or epigenetic instability (14). The diagnostic challenge is compounded by inter-laboratory variability and lack of standardization in semen assessment. Even within the framework of WHO reference values, men with borderline parameters may achieve natural conception, whereas men with apparently normal profiles may fail to conceive (15). This paradox underscores that semen analysis is descriptive but not mechanistic, failing to capture the molecular underpinnings of fertility potential. Similarly, endocrine evaluation may identify hypogonadism or pituitary disorders but remains uninformative for men with idiopathic infertility, where gonadotropins and testosterone are typically within reference ranges (16). Moreover, the reliance on exclusionary diagnosis, labelling patients as idiopathic after ruling out known causes, limits clinical decision-making. Such categorization provides little guidance for treatment, contributing to the inconsistent outcomes of antioxidant trials, which often fail due to patient heterogeneity, lack of redox stratification, inappropriate dosing, and the inclusion of men without demonstrable oxidative imbalance (3, 11). In this context, redox endophenotyping offers a theoretical framework to improve therapeutic efficacy by identifying patients most likely to benefit from antioxidant intervention while avoiding overtreatment and reductive stress in others.

Importantly, idiopathic infertility is not a true diagnosis but a reflection of the diagnostic gap in current practice. Thus, the definitional and diagnostic challenges of IMI demand a paradigm shift. Moving toward molecular phenotyping, particularly redox-based profiling, could enable reclassification of this heterogeneous group into biologically meaningful subtypes. Such an approach may transform idiopathic infertility from a diagnosis of exclusion into a mechanistically informed category, enabling targeted interventions and improved reproductive outcomes.

### 2.2 Limitations and current diagnostic markers

**Figure 1:**
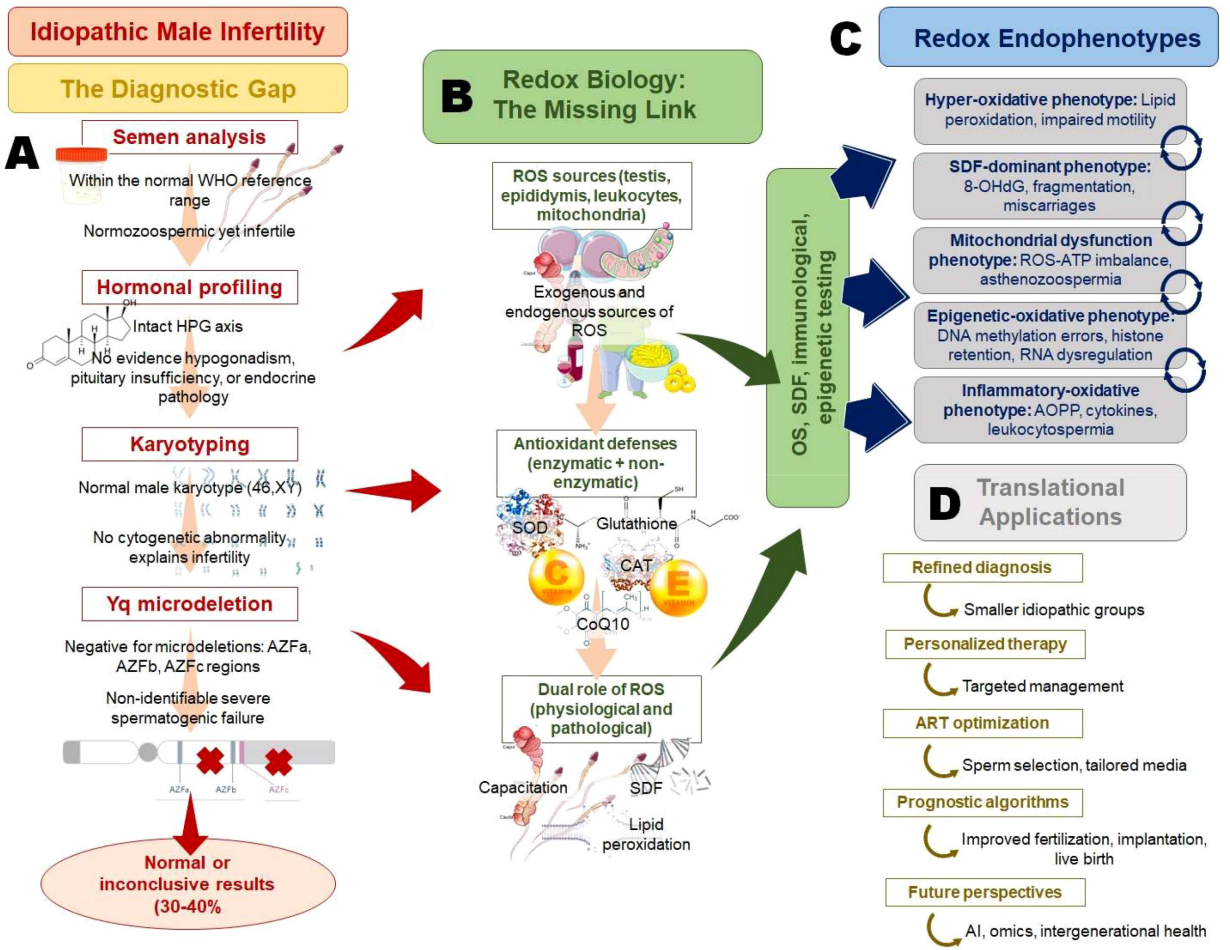
From diagnostic gaps to redox endophenotypes: a precision framework for idiopathic male infertility. Idiopathic male infertility arises from the limitations of conventional diagnostics, **(A)** where semen analysis, hormonal profiling, karyotyping, and Yq microdeletion testing often yield normal or inconclusive results. Redox biology provides the missing mechanistic link **(B)** with ROS exerting dual physiological and pathological roles depending on antioxidant balance. Stratification into distinct redox endophenotypes **(C)** provides a refined framework for explaining idiopathic cases. These insights enable translational applications **(D)** such as mechanistically guided diagnosis, personalized therapy, optimized ART strategies, and prognostic algorithms, while paving the way for integration with omics, artificial intelligence, and intergenerational health perspectives.

The clinical evaluation of male infertility has long relied on a triad of diagnostic markers: semen parameters, hormonal profiles, and genetic screening (**Figure 1**). While these tools remain the cornerstone of clinical practice, they are inherently limited in their ability to resolve the mechanistic causes underlying idiopathic infertility (17). Semen analysis, guided by WHO criteria, remains the first-line diagnostic test, assessing sperm concentration, motility, and morphology (15). However, these parameters offer only a gross estimate of reproductive potential and lack predictive value for fertilization competence (10). For example, two men with comparable semen profiles may display dramatically different ART outcomes, while a proportion of normozoospermic men remain infertile despite apparently normal semen quality. These observations reflect the inability of conventional semen parameters to account for oxidative damage, mitochondrial dysfunction, and epigenetic alterations that compromise sperm function. Hormonal profiling, including follicle-stimulating hormone (FSH), luteinizing hormone (LH), prolactin, and testosterone, aids in diagnosing endocrine dysfunction such as hypogonadotropic hypogonadism or primary testicular failure (18). However, in idiopathic infertility, endocrine parameters are typically within normal ranges (19), offering little discriminatory power. Subtle dysregulation of intratesticular steroidogenesis, paracrine signaling, or receptor sensitivity remains undetected, further reinforcing the inadequacy of routine hormonal assays (2). Genetic testing, particularly karyotyping and Y-chromosome microdeletion analysis, has identified important etiologies, such as Klinefelter syndrome and AZF deletions (20). Yet, the diagnostic yield of these tests is relatively low, collectively accounting for only 5-10% of infertile men. Moreover, conventional genetic assessments fail to capture epigenetic perturbations, mitochondrial genome instability, or polymorphisms affecting oxidative metabolism, which may significantly contribute to infertility risk (21).

A key limitation across these markers is their static, descriptive nature, which fails to reflect the dynamic processes that influence sperm maturation and function. Furthermore, the lack of integration between clinical, biochemical, and molecular domains results in fragmented assessment, with many men ultimately classified as idiopathic. Emerging tools, such as sperm DNA fragmentation assays and OS biomarkers, have begun to bridge this gap, but challenges of standardization, reproducibility, and clinical adoption remain (22, 23). Ultimately, reliance on conventional markers perpetuates the diagnostic ambiguity surrounding IMI. This limitation underscores the need for advanced, mechanism-driven diagnostics, such as redox endophenotyping, to move beyond descriptive classification and enable personalized therapeutic strategies.

### 2.3 Burden of ‘idiopathic’ category in clinical practice and ART outcomes

The persistence of the idiopathic infertility category poses significant clinical and psychosocial challenges. For patients, the absence of a clear diagnosis often translates into uncertainty, anxiety, and dissatisfaction, as couples are left without an explanation for their reproductive difficulties (24). For clinicians, idiopathic cases represent a therapeutic dilemma, where treatment decisions are frequently empirical rather than evidence-based (25). From a clinical standpoint, idiopathic infertility accounts for up to 40% of male infertility cases, reflecting the inadequacy of existing diagnostic frameworks. This large residual category complicates treatment stratification, as patients with fundamentally different pathophysiologies are grouped together under the same label (2). As a result, empirical therapies such as antioxidant supplementation or empiric intrauterine insemination (IUI) are often employed, with highly variable and often disappointing outcomes (26). The lack of mechanistic clarity hampers the ability to identify which men may benefit from targeted interventions or require escalation to ART. In the context of ART, idiopathic infertility significantly impacts decision-making and outcomes (11). Men categorized as idiopathic are often fast-tracked to *in vitro* fertilization (IVF) or intracytoplasmic sperm injection (ICSI), bypassing attempts at natural conception or less invasive interventions (27). While ICSI has revolutionized treatment, its use in idiopathic infertility raises concerns, as it addresses the symptom, fertilization failure, without resolving the underlying male factor (28). Moreover, evidence suggests that sperm with unresolved oxidative or genetic defects may compromise embryo development, implantation, and offspring health, even when ICSI is successful (29, 30). This raises long-term concerns about the intergenerational impact of unresolved IMI (31). Economically, the burden of idiopathic infertility is substantial. Couples often undergo repeated cycles of ART, incurring high financial and emotional costs without guaranteed success. Furthermore, the reliance on trial-and-error approaches contributes to inefficiencies in healthcare delivery, diverting resources from more targeted and effective strategies (32).

The persistence of the idiopathic category also limits progress in reproductive research. By failing to delineate mechanistic subgroups, valuable opportunities for biomarker discovery, therapeutic innovation, and precision medicine are missing. Consequently, idiopathic infertility represents not only a clinical burden but also a scientific bottleneck. Addressing this burden requires a shift from exclusionary diagnosis toward mechanistic stratification. Redox endophenotyping, by identifying OS-driven subtypes of infertility, offers a promising pathway to reduce the size of the idiopathic category, refine treatment strategies, and ultimately improve ART outcomes and reproductive health.

### 2.4 The need for endophenotyping as a bridge between phenotype and genotype

The concept of endophenotyping offers a valuable framework for resolving the diagnostic ambiguity associated with IMI. Endophenotypes are intermediate, quantifiable biological traits that link observable clinical phenotypes to underlying genetic and molecular mechanisms (33, 34). By capturing functional disruptions that lie between genotype and phenotype, endophenotyping offers an avenue to move beyond descriptive diagnostics and toward mechanistic classification (34). In male infertility, the phenotype is typically defined by semen parameters, while the genotype is probed through karyotyping, Y-chromosome microdeletion analysis, or targeted genetic panels (35). However, the gap between these levels of assessment remains vast. Men with similar semen profiles may harbour distinct molecular dysfunctions, while those with comparable genetic backgrounds may manifest variable clinical outcomes. This discordance reflects the influence of intermediate processes such asOS, mitochondrial dysfunction, and epigenetic regulation, which remain invisible to current diagnostic practices (2, 4).

Redox biology provides a compelling entry point for developing endophenotypes (4). OS represents a convergent pathway through which genetic susceptibilities, environmental exposures, and lifestyle factors influence sperm function (36–38). For example, variants in antioxidant defence genes, exposure to environmental toxins, or chronic inflammation may all converge to generate a hyper-oxidative phenotype (37). Similarly, defects in mitochondrial bioenergetics, chromatin remodeling, or epigenetic programming may be unified under redox-sensitive mechanisms (39–41). Such mechanistic clustering provides a more precise framework for stratifying patients than broad phenotypic categories like normozoospermia or oligoasthenoteratozoospermia (42). By integrating redox-based endophenotypes into diagnostic practice, clinicians may be able to identify subgroups of men who would benefit from targeted antioxidant therapy, mitochondrial modulators, or epigenetic interventions. Moreover, endophenotyping may enhance prognostication in ART by predicting which sperm populations are most likely to yield healthy embryos and viable pregnancies (42). Importantly, endophenotyping also offers a bridge to translational research. By linking molecular pathways to clinical outcomes, it facilitates biomarker discovery, therapeutic innovation, and personalized medicine approaches. Advances in omics technologies, coupled with computational modeling, can further refine endophenotypic classifications, enabling multidimensional integration of redox, genetic, and exposomic data (43, 44). Thus, the application of endophenotyping represents a paradigm shift in the study of IMI. By operationalizing the intermediate space between genotype and phenotype, it promises to transform idiopathic infertility from a diagnosis of exclusion into a set of mechanistically defined subgroups. This transition is essential for achieving precision andrology and for reducing the persistent reliance on the ambiguous ‘idiopathic’ label.

## 3 Redox biology of the male reproductive system

### 3.1 Sources of ROS in testis, epididymis, and seminal plasma

ROS generation is an inevitable outcome of cellular metabolism, and in the male reproductive tract, they play a dual role in sustaining physiological processes and driving pathological damage (7) (**Figure 1**). The testes, epididymis, and seminal plasma represent distinct yet interconnected microenvironments where ROS are produced by intrinsic and extrinsic sources (4, 6). Within the testes, the mitochondria of germ cells and Sertoli cells are primary sites of ROS production via electron leakage from complexes I and III of the electron transport chain (45). The high proliferative activity of germ cells, coupled with intensive chromatin remodeling during spermatogenesis, creates conditions prone to oxidative imbalance (46). Furthermore, Leydig cells contribute to ROS generation during steroidogenesis, as enzymatic reactions involving cytochrome P450 produce superoxide anions and hydrogen peroxide (47). Disruption of antioxidant defense within this milieu can result in germ cell apoptosis, meiotic arrest, or impaired testosterone biosynthesis, ultimately compromising spermatogenesis (48). In the epididymis, ROS production is enhanced by the presence of leukocytes, particularly macrophages and neutrophils, which are recruited during subclinical inflammation or infection (49). Epididymal epithelial cells also contribute to redox activity through NADPH oxidase isoforms, which regulate luminal redox homeostasis essential for sperm maturation (50). However, pathological activation of these enzymes leads to excessive ROS release, altering epididymal secretions that are critical for sperm membrane remodeling, motility acquisition, and fertilizing potential (51). Seminal plasma represents the final compartment where spermatozoa encounter OS. Sperm themselves generate ROS, primarily through mitochondrial activity and membrane-bound NADPH oxidases. Interestingly, immature spermatozoa with residual cytoplasm are major contributors to ROS due to their high glucose-6-phosphate dehydrogenase activity, which fuels NADPH-dependent oxidase pathways (52). Additionally, activated leukocytes in semen can produce ROS levels up to 1000-fold higher than those of spermatozoa, rendering leukocytospermia a clinically significant contributor to oxidative infertility (53).

External factors such as environmental toxins, smoking, varicocele, and metabolic disorders further exacerbate ROS generation across these compartments (5). Importantly, ROS produced in the testes may prime spermatozoa for damage that is amplified in the epididymal and seminal environments. This cumulative oxidative burden highlights the necessity of compartment-specific analysis in understanding male infertility (5). In idiopathic cases, conventional diagnostics fail to detect these localized redox disturbances (4). Thus, characterizing ROS sources across the reproductive tract is critical for identifying mechanistic subgroups of patients, paving the way for redox endophenotypes that transcend the idiopathic label.

### 3.2 Antioxidant defense systems: enzymatic and non-enzymatic layers

The male reproductive tract is equipped with a sophisticated antioxidant defense system designed to maintain redox equilibrium. These defenses encompass enzymatic and non-enzymatic layers that operate synergistically to neutralize ROS and preserve sperm function (54, 55). Disruption of this finely tuned network is a central event in the pathogenesis of OS-related infertility. Enzymatic antioxidants form the first line of defense. Superoxide dismutase (SOD) catalyzes the dismutation of superoxide anions into hydrogen peroxide, which is further detoxified by catalase and glutathione peroxidase (GPx). The importance of SOD isoforms is evident from their localization: mitochondrial Mn-SOD safeguards oxidative phosphorylation, while Cu/Zn-SOD protects cytoplasmic and extracellular compartments (56). GPx, particularly GPx4, plays a dual role as both an antioxidant enzyme and a structural component of sperm chromatin, highlighting its reproductive relevance (57). Deficiency or inactivation of these enzymes results in unchecked ROS accumulation, lipid peroxidation, and impaired motility. Non-enzymatic antioxidants complement enzymatic defenses by scavenging free radicals and reinforcing redox homeostasis (58). Reduced glutathione (GSH) serves as a key thiol donor, participating in detoxification reactions catalyzed by glutathione S-transferase (59). Vitamins C and E provide additional layers of protection: ascorbate neutralizes aqueous ROS, while α-tocopherol prevents lipid peroxidation of sperm membranes rich in polyunsaturated fatty acids (60). Micronutrients such as selenium, zinc, and coenzyme Q10 further contribute by supporting enzymatic activity or directly scavenging radicals (61–63).

Seminal plasma acts as a reservoir of both enzymatic and non-enzymatic antioxidants, creating a protective buffer for ejaculated spermatozoa (58). However, sperm themselves possess limited intrinsic antioxidant capacity due to the minimal cytoplasmic volume, making them highly dependent on external redox defenses (64). This vulnerability is exacerbated in idiopathic infertility, where subtle deficiencies in seminal antioxidants may remain undetected by conventional assays. The balance between enzymatic and non-enzymatic layers is delicate; excess supplementation of non-enzymatic antioxidants may induce ‘reductive stress’ (RS), impairing physiological ROS-dependent processes (65, 66). Thus, antioxidant defenses must be considered not only in terms of capacity but also in dynamic regulation. Critically, idiopathic infertility may reflect hidden defects in this antioxidant system. For instance, polymorphisms in GPx4 or SOD genes, micronutrient deficiencies, or impaired recycling of antioxidant pools can predispose individuals to a redox imbalance without overt clinical markers (67). Integrating antioxidant profiling into diagnostic practice could reveal such hidden susceptibilities, thereby refining the classification of idiopathic cases into biologically meaningful endophenotypes.

### 3.3 Physiological role of ROS in sperm capacitation, acrosome reaction, and fertilization

While often pathologized, ROS are indispensable signaling molecules in male reproduction, particularly during the processes of capacitation, acrosome reaction, and fertilization (7). Their role illustrates the paradox of ROS biology: essential at physiological concentrations but deleterious when unchecked (68). Capacitation, the functional maturation of spermatozoa in the female reproductive tract, requires ROS-mediated activation of intracellular signaling cascades (69). Superoxide and hydrogen peroxide modulate tyrosine phosphorylation of sperm proteins, facilitating changes in membrane fluidity and ion fluxes (70). These events prime spermatozoa for hyperactivated motility, a prerequisite for navigating the oviductal environment. Inhibition of ROS generation during capacitation results in defective tyrosine phosphorylation and impaired fertilization capacity, underscoring their physiological necessity (7). The acrosome reaction, an exocytotic event enabling sperm penetration through the zona pellucida, is also ROS-dependent (71). Hydrogen peroxide enhances calcium influx and activates signaling pathways that trigger membrane fusion and acrosomal enzyme release (72). ROS additionally regulate actin dynamics, ensuring proper cytoskeletal remodeling during this process (73). Thus, controlled ROS generation serves as a molecular switch for acrosomal exocytosis.

Beyond these events, ROS contribute to sperm-oocyte interaction and fusion (7). Mild oxidative modifications of sperm surface proteins may increase zona pellucida binding, while redox signaling influences mitochondrial activity, ensuring adequate ATP supply for sustained motility (74). Furthermore, ROS participate in chromatin decondensation during fertilization, facilitating male pronucleus formation (68). However, the fine line between physiological and pathological ROS levels is easily disrupted. Excessive ROS impair capacitation, induces premature acrosome reactions, and damage DNA integrity, whereas insufficient ROS blunts the signaling required for fertilization competence (4, 5). This narrow therapeutic window poses a challenge for clinical interventions aimed at modulating redox balance. In idiopathic infertility, subtle disturbances in ROS-mediated signaling may manifest as functional impairments that evade detection by conventional semen analysis (4). For instance, sperm may display normal motility yet fail to undergo proper capacitation or acrosome reaction due to redox dysregulation. Identifying such dysfunctions through functional assays could help stratify idiopathic cases into ROS-related endophenotypes, providing mechanistic insight and guiding therapeutic approaches.

### 3.4 Oxidative stress versus reductive stress: defining the redox balance in sperm function

The concept of redox balance is central to understanding sperm physiology and pathology. While OS, defined as excess ROS relative to antioxidant capacity, is widely recognized as a driver of male infertility, the emerging concept of RS adds complexity to this paradigm (5, 65). Both extremes of the redox spectrum can compromise sperm function, highlighting the need to define optimal redox homeostasis rather than simply minimizing ROS (66). OS damages spermatozoa through lipid peroxidation, protein carbonylation, and DNA fragmentation, leading to reduced motility, impaired acrosome reaction, and compromised fertilization potential (51). Clinical studies consistently demonstrate elevated markers of oxidative damage in infertile men, with strong associations to poor ART outcomes (75). However, interventions aimed solely at reducing ROS often yield inconsistent results, suggesting that indiscriminate antioxidant therapy may disrupt physiological redox signaling (6). RS, characterized by an excessive antioxidant environment, can suppress the ROS-dependent pathways necessary for capacitation, hyperactivation, and acrosome reaction (65). For example, over-supplementation with vitamins C and E or glutathione precursors may scavenge ROS to sub-physiological levels, blunting essential signaling cascades. This phenomenon, termed the ‘antioxidant paradox,’ underscores the risk of empirically prescribing antioxidants in idiopathic infertility without mechanistic stratification (48, 76).

Defining the redox balance thus requires precise assessment of both oxidative and reductive states (8). Emerging assays such as oxidation-reduction potential (ORP) measurements offer integrative insights into global redox status, bridging the gap between fragmented biomarkers (37). Yet, challenges of standardization, threshold determination, and clinical interpretation remain. For IMI, the critical issue lies in recognizing that not all patients suffer from OS. Some may exhibit reductive imbalance due to lifestyle factors, excessive supplementation, or intrinsic metabolic traits (66). Without distinguishing these states, therapeutic interventions risk exacerbating dysfunction rather than restoring fertility. The conceptual shift from ‘oxidative damage’ to ‘redox imbalance’ provides a foundation for redox endophenotyping. By categorizing patients into hyper-oxidative, reductive, or mixed phenotypes, clinicians can tailor interventions, avoid the pitfalls of empirical antioxidant therapy, and improve ART outcomes. Ultimately, the recognition of both oxidative and RS reframes idiopathic infertility as a disorder of redox dysregulation, opening new avenues for precision diagnostics and targeted therapeutics.

## 4 Mechanistic pathways linking oxidative stress to male infertility

### 4.1 Lipid peroxidation of sperm membranes and consequences for motility and fusion

Spermatozoa are uniquely vulnerable to lipid peroxidation owing to their plasma membranes being enriched with polyunsaturated fatty acids (PUFAs), particularly docosahexaenoic acid (DHA) (77). While these lipids confer fluidity, which is essential for capacitation and fusion with the oocyte, they also contain multiple double bonds that are highly susceptible to oxidative attack. When ROS exceed the buffering capacity of antioxidants, lipid peroxidation is initiated through chain reactions involving hydroxyl radicals and peroxyl intermediates (78). The biochemical cascade of lipid peroxidation generates reactive aldehydes, including malondialdehyde (MDA) and 4-hydroxy-2-nonenal (4-HNE). These secondary products are not merely markers of oxidative injury but active disruptors of sperm function (78). By forming covalent adducts with membrane proteins, they impair ion channel activity, signaling pathways, and membrane fusion processes critical for fertilization (79). Furthermore, aldehyde adduction can alter mitochondrial membrane potential, reducing ATP supply necessary for hyperactivated motility (80).

The clinical relevance of lipid peroxidation is underscored by strong associations between elevated seminal MDA or isoprostane levels and reduced sperm motility, viability, and fertilization capacity (78). Studies demonstrate that men with idiopathic infertility often harbour elevated lipid peroxidation despite normal semen parameters, suggesting that conventional diagnostics miss these functional impairments (81). Importantly, sperm motility is highly sensitive to lipid peroxidation because flagellar beating requires tightly regulated calcium flux and ATP hydrolysis, both of which are disrupted by peroxidative damage (82). Fusion events are similarly compromised. Lipid peroxidation alters membrane fluidity, preventing the physiological reorganization of lipid rafts and the exposure of fusogenic proteins during acrosome reaction (83). This results in diminished sperm-oocyte binding and fertilization failure even in normozoospermic men. Such cases are often categorized as idiopathic infertility, yet the mechanistic driver is oxidative lipid injury. In ART, lipid peroxidation also has implications. Sperm selected for ICSI may retain peroxidative damage, transmitting defective membranes and aldehyde adducts into the oocyte cytoplasm (84). This can impair pronuclear development, embryo quality, and implantation outcomes. Cryopreservation further amplifies lipid peroxidation by inducing oxidative bursts during freeze-thaw cycles, aggravating damage in idiopathic cases (85).

As discussed earlier, therapeutic interventions targeting lipid peroxidation, such as supplementation with vitamin E, coenzyme Q10, or polyphenols, have shown variable outcomes, reflecting the need for patient stratification (60, 63). Empirical antioxidant therapy may rescue some patients while exacerbating RS in others (65). This variability underscores the rationale for a redox endophenotype approach, specifically, identifying a ‘hyper-oxidative lipid peroxidation phenotype’ among idiopathic men to guide therapy. Thus, lipid peroxidation represents a pivotal mechanism linking ROS imbalance to impaired motility and fertilization, accounting for a subset of idiopathic infertility cases (78, 83). Its recognition and quantification could transform a vague idiopathic diagnosis into a defined, mechanistically driven endophenotype with direct therapeutic implications.

### 4.2 Protein oxidation, nitration, and impairment of mitochondrial function

Proteins are major targets of oxidative and nitrosative stress in spermatozoa due to the abundance of susceptible amino acid residues such as cysteine, tyrosine, methionine, and tryptophan (86). Oxidative modifications, including carbonylation, disulfide crosslinking, and nitrotyrosine formation, can profoundly alter protein structure and function (87). Given the specialized proteome of sperm, encompassing enzymes for energy metabolism, ion transporters, and structural proteins for motility, oxidative protein damage has disproportionate effects on fertility potential (88).

One critical pathway involves nitration of tyrosine residues by peroxynitrite, a reactive species generated through the reaction of nitric oxide with superoxide (89). Nitrotyrosine modification of axonemal proteins impairs flagellar motility, while nitration of mitochondrial enzymes disrupts oxidative phosphorylation (86). Similarly, oxidation of cysteine residues in ATP synthase or adenine nucleotide translocase compromises mitochondrial ATP production (90). Since sperm motility is heavily dependent on mitochondrial bioenergetics, such alterations manifest as asthenozoospermia even when sperm counts and morphology are normal (91). Mitochondria themselves are both sources and victims of OS. ROS leakage from the electron transport chain damages mitochondrial DNA and proteins, perpetuating a vicious cycle of dysfunction (39). The decline in mitochondrial membrane potential leads to impaired ATP synthesis, increased ROS leakage, and activation of intrinsic apoptotic pathways. In spermatozoa, this translates into defective motility, premature capacitation, and increased susceptibility to apoptosis-like changes (39, 45).

Oxidative modifications of proteins also impair fertilization processes. For instance, the oxidation of acrosomal enzymes, such as acrosin, reduces the zona pellucida penetration capacity (92). Similarly, oxidative damage to surface proteins involved in sperm-oocyte recognition diminishes binding affinity, resulting in fertilization failure despite apparently normal semen parameters (29). Clinical studies consistently demonstrate elevated levels of oxidized proteins and nitrotyrosine in semen of infertile men (86). In idiopathic infertility, such damage may be the hidden molecular defect explaining poor ART outcomes. Notably, oxidative protein modifications are not readily reversible, unlike reversible thiol oxidation, which underscores their diagnostic significance (87). Therapeutically, interventions targeting mitochondrial redox status, such as mitochondrial-targeted antioxidants (e.g., MitoQ, SkQ1), have shown promise in preclinical studies (93, 94). However, clinical translation is hindered by the inability to stratify patients who truly exhibit oxidative protein damage. This again reinforces the need for a ‘mitochondrial dysfunction endophenotype’ within idiopathic infertility, which would enable targeted therapies and avoid empirical approaches. Thus, oxidative and nitrosative modifications of sperm proteins represent a major pathway linking redox imbalance to infertility (87, 89). Their role in compromising mitochondrial function and motility situates them as a central mechanism underlying idiopathic cases, warranting integration into diagnostic frameworks for mechanistic classification.

### 4.3 Oxidative DNA damage, sperm chromatin remodeling, and protamination defects

The integrity of sperm DNA is fundamental to reproductive success, ensuring faithful transmission of genetic information to the embryo (95). OS is one of the most significant threats to sperm genomic stability, inducing a spectrum of DNA lesions ranging from single- and double-strand breaks to base modifications (5). Among these, 8-hydroxy-2′-deoxyguanosine (8-OHdG) is the most widely studied biomarker of oxidative DNA damage, strongly associated with infertility and adverse ART outcomes (83). Sperm chromatin architecture renders DNA particularly susceptible to damage. During spermiogenesis, histones are largely replaced by protamines, resulting in a highly compact chromatin structure resistant to damage (96). However, incomplete protamination, common in infertile men, leaves DNA regions susceptible to ROS attack (97). Protamine deficiency also impairs disulfide crosslinking, weakening nuclear stability (98). As a result, the DNA fragmentation index (DFI) is often elevated in idiopathic infertility despite normal semen parameters, highlighting cryptic defects undetected by routine diagnostics (99).

Oxidative DNA damage carries both immediate and long-term consequences. At the fertilization level, DNA strand breaks can impair paternal pronucleus formation, triggering embryonic arrest (100). At the intergenerational level, oxidative lesions may evade repair during early embryogenesis, leading to mutations, imprinting errors, and increased risk of childhood disorders (101). This intergenerational impact raises significant ethical and clinical concerns regarding the use of sperm with oxidative DNA damage in ART. Chromatin remodeling defects further exacerbate susceptibility. Retention of histones in promoter regions of developmentally critical genes, coupled with oxidative modifications, disrupts epigenetic programming (102). In addition, OS interferes with topoisomerase activity and chromatin condensation, further destabilizing the sperm genome (103). These subtle yet profound alterations are rarely assessed in clinical practice, contributing to the idiopathic label.

Clinical evidence links high levels of sperm DNA fragmentation with poor ART outcomes, including reduced fertilization rates, impaired blastocyst development, and recurrent pregnancy loss (104, 105). While assays such as TUNEL, Comet, and SCSA provide valuable insights, they do not discriminate between oxidative and non-oxidative DNA damage, which limits therapeutic guidance (106, 107). Targeted approaches, including antioxidant therapy, lifestyle modification, and sperm selection techniques (e.g., microfluidic sorting, magnetic-activated cell sorting), aim to mitigate DNA damage (22). However, their inconsistent success underscores the heterogeneity of underlying mechanisms. Identifying a ‘DNA damage-dominant redox endophenotype’ could provide clarity, allowing clinicians to apply precision interventions and refine ART protocols. Thus, oxidative DNA damage and protamination defects represent a critical mechanistic bridge between ROS imbalance and idiopathic infertility. Their recognition as a distinct endophenotype could transform diagnostic categorization, therapeutic decision-making, and ultimately patient outcomes.

### 4.4 ROS-mediated epigenetic modifications: DNA methylation, histone retention, and sperm RNAs

Beyond structural DNA damage, OS profoundly influences the sperm epigenome, affecting not only fertility but also transgenerational health (108). Epigenetic modifications, including DNA methylation, histone retention, and non-coding RNAs, are exquisitely sensitive to ROS levels, making them central to the mechanistic pathways linking redox imbalance to idiopathic infertility (109).

DNA methylation is a critical epigenetic mechanism regulating gene expression and imprinting (110). OS perturbs DNA methyltransferase activity and induces oxidation of 5-methylcytosine to 5-hydroxymethylcytosine, leading to abnormal methylation patterns (111). Aberrant methylation of imprinted loci such as H19 and IGF2 has been observed in infertile men, correlating with poor embryo quality (112, 113). These defects may persist in offspring, predisposing them to metabolic and neurodevelopmental disorders, underscoring the intergenerational consequences of sperm redox imbalance. Histone retention provides another vulnerable axis. While protamination compacts most of the sperm genome, ~5-10% of histones are retained at regulatory regions (114). OS disrupts histone-protamine exchange, leading to excessive histone retention enriched with oxidative adducts (96). This perturbs chromatin accessibility, impairing transcriptional reprogramming post-fertilization. Clinical studies demonstrate increased histone retention in idiopathic infertility, suggesting a mechanistic contribution to otherwise unexplained cases (115). Sperm RNAs, including microRNAs (miRNAs), piRNAs, and long non-coding RNAs, are also sensitive to oxidative alterations (116). ROS can degrade small RNAs or alter their expression profiles, thereby disrupting paternal contributions to early embryogenesis. For example, dysregulated sperm miRNAs under OS have been linked to defective embryo cleavage and implantation failure (117). Since sperm RNAs are critical for epigenetic reprogramming, their disruption provides a plausible mechanism for idiopathic infertility and adverse ART outcomes (118). Thus, these epigenetic alterations extend the impact of OS beyond immediate sperm dysfunction to long-term reproductive consequences. Importantly, conventional diagnostics do not assess sperm epigenetics, perpetuating the idiopathic label. Incorporating redox-sensitive epigenetic biomarkers could identify an ‘epigenetic-oxidative endophenotype,’ enabling stratification of patients at risk of both infertility and transgenerational disease. Therapeutically, interventions remain experimental, including epigenetic editing, dietary methyl donors, and antioxidant therapies. However, indiscriminate supplementation risks exacerbating reductive imbalance. A precision medicine approach, guided by epigenetic profiling, holds promise for restoring sperm epigenome integrity and reducing the burden of idiopathic infertility.

### 4.5 Crosstalk between inflammation, infection, and oxidative stress in ‘unexplained’ infertility

Inflammation and infection are powerful drivers of OS in the male reproductive tract, and their interplay represents a critical yet underrecognized contributor to idiopathic infertility (119, 120). Even in the absence of overt clinical symptoms, subclinical inflammation or occult infection can generate sustained ROS production, leading to sperm dysfunction. Leukocytes are central to this crosstalk. Activated neutrophils and macrophages release superoxide, hydrogen peroxide, and myeloperoxidase-derived radicals at levels up to 1000 times greater than spermatozoa (49, 53). While intended for pathogen clearance, this ROS surge spills into the seminal environment, damaging sperm membranes, proteins, and DNA. Chronic prostatitis, epididymitis, and subclinical genital tract infections are frequent but often overlooked sources of such OS (120). Inflammatory mediators further amplify redox imbalance. Cytokines such as TNF-α, IL-6, and IL-1β upregulate NADPH oxidases in reproductive tissues, perpetuating ROS production (121). Simultaneously, inflammation depletes antioxidant defenses, creating a feed-forward loop of oxidative injury (48). The result is a hostile microenvironment where spermatozoa, already deficient in intrinsic antioxidant capacity, suffer irreversible damage. Crucially, this inflammation-OS axis often manifests without overt alterations in semen parameters, leading to a diagnosis of idiopathic infertility (48). For instance, men with normozoospermia but elevated seminal leukocytes or cytokines may exhibit high DNA fragmentation and poor ART outcomes yet remain categorized as idiopathic under conventional frameworks.

Infection-driven OS also carries transgenerational implications. Pathogens such as Chlamydia trachomatis and Ureaplasma urealyticum induce oxidative DNA damage and epigenetic alterations in sperm, which may influence embryo development (122). Persistent infections may also induce autoimmunity against sperm antigens, compounding infertility risk (123). Clinically, the challenge lies in distinguishing between physiological immune surveillance and pathological inflammation. Conventional semen analysis does not assess inflammatory markers, while routine cultures may miss intracellular or biofilm-forming pathogens. This diagnostic gap perpetuates the use of the idiopathic label. Incorporating assays for OS, seminal cytokines, and molecular pathogen detection could uncover a distinct ‘inflammatory-oxidative endophenotype.’ Therapeutic strategies must balance antimicrobial treatment, anti-inflammatory interventions, and antioxidant support to achieve optimal outcomes. While antibiotics resolve acute infections, persistent inflammatory OS may require adjunctive therapies (54). Emerging evidence suggests the benefits of targeted antioxidants, such as N-acetylcysteine, alongside anti-inflammatory nutraceuticals, though patient stratification is essential to avoid overtreatment (124). Therefore, the crosstalk between inflammation, infection, and OS provides a unifying mechanism for many cases of ‘unexplained’ infertility. Recognizing this interplay as a mechanistic endophenotype offers opportunities for targeted diagnostics, improved therapy, and reduction of the idiopathic burden in clinical practice.

## 5 Clinical assessment of redox status in semen

### 5.1 Conventional oxidative stress assays

Conventional assays for OS in semen have long served as investigative tools in andrology (**Table 1**), aiming to quantify redox imbalance by either measuring oxidative damage or antioxidant capacity (8). Among these, malondialdehyde (MDA), total antioxidant capacity (TAC), GSH, catalase, and SOD are the most widely employed, each capturing a different aspect of the oxidative landscape (8). While valuable, these markers have limitations in terms of sensitivity, specificity, and translational utility, which contribute to the persistence of the idiopathic infertility label. MDA, a byproduct of lipid peroxidation, is one of the earliest and most widely used biomarkers of OS in semen (83). Elevated MDA levels correlate strongly with impaired motility, abnormal morphology, and increased DNA fragmentation (78, 83). Clinical studies consistently demonstrate higher MDA levels in infertile men compared with fertile controls (79). However, MDA quantification is subject to variability across assays (e.g., the thiobarbituric acid-reactive substances test), which lacks specificity because it reacts with other aldehydes and carbohydrates. This methodological limitation reduces its clinical reliability (125). TAC provides a holistic estimate of the seminal antioxidant reserve by integrating enzymatic and non-enzymatic defenses. It is useful in distinguishing between fertile and infertile men, with reduced TAC commonly reported in idiopathic infertility (126). Nonetheless, TAC values are influenced by diet, supplementation, and lifestyle, making it difficult to interpret them in isolation (127). Furthermore, TAC fails to differentiate between specific enzymatic deficiencies or excessive antioxidant supplementation (66), both of which have clinical relevance.

**Table 1:** Clinical assessment of redox status in semen: conventional, Integrative, and emerging biomarkers.

Biomarker/assay	Type of redox assessment	Strengths	Limitations	Clinical utility in IMI	References
MDA (TBARS assay)	Lipid peroxidation	Widely studied; correlates with motility	Non-specific; assay variability	Marker of hyper-oxidative phenotype	Aitken 1995; Pannu 2022 (78, 83)
TAC (Total Antioxidant Capacity)	Antioxidant reserves	Holistic; easy to measure	Influenced by diet/lifestyle; not pathway-specific	Screening of seminal antioxidant status	Gupta 2021 (126)
ORP (MiOXSYS system)	Global redox balance	Rapid, reproducible, integrative	Lacks mechanistic specificity	Identifies hidden hyper-oxidative states in normozoospermic IMI	Agarwal 2017; Majzoub 2018 (133, 135)
8-OHdG	Oxidative DNA damage	Strongly linked to ART outcomes	Requires standardization; cost	Defines DNA damage-dominant phenotype	Mukheef 2022 (142)
Isoprostanes	Lipid peroxidation	Stable, specific markers	Technical complexity	Gold-standard for oxidative lipid injury	Signorini 2020 (145)
AOPP	Protein oxidation/ inflammation-linked	Reflects leukocyte-derived ROS	Less validated in large cohorts	Identifies inflammatory-oxidative phenotype	Janiszewska 2022 (137)

GSH, a tripeptide thiol, represents a central non-enzymatic antioxidant in seminal plasma (57). Its concentration reflects cellular redox status and capacity to neutralize hydrogen peroxide through GPx. Reduced seminal GSH is associated with increased lipid peroxidation and sperm DNA fragmentation (128). However, routine measurement is technically challenging, requiring high-performance liquid chromatography or enzymatic cycling assays, which are not standardized across laboratories (129). Catalase and SOD represent enzymatic defenses against ROS (8). SOD catalyzes the dismutation of superoxide radicals into hydrogen peroxide, while catalase detoxifies hydrogen peroxide into water and oxygen (130). Reduced activities of these enzymes have been reported in idiopathic infertile men (58), correlating with poor motility and increased oxidative markers. Yet, enzyme activity assays are highly sensitive to sample handling, storage, and assay conditions, resulting in poor reproducibility. Overall, conventional OS assays provide useful insights into redox balance but are limited by methodological variability, lack of standardization, and poor integration into routine diagnostics. They often serve as research tools rather than as markers for clinical decision-making. Importantly, these assays capture fragments of the oxidative landscape, but do not offer a comprehensive, real-time measure of global redox status. As such, they contribute to the persistence of the idiopathic infertility category, underscoring the need for more integrative and standardized redox biomarkers (8).

### 5.2 Oxidation-reduction potential as an integrative marker

ORP has emerged as a novel, integrative biomarker for assessing redox status in semen, addressing many of the limitations of conventional assays (37). Unlike single-parameter tests that measure either oxidants or antioxidants, ORP quantifies the balance between total oxidants and reductants in a single, real-time measurement, expressed in millivolts. This approach provides a comprehensive assessment of OS, bridging the gap between fragmented biomarkers and their functional relevance (131). The introduction of the MiOXSYS system has standardized ORP measurement in clinical andrology. ORP values are normalized to sperm concentration, yielding a static ORP (sORP) index that accounts for differences in sperm load (132). Elevated sORP values have been consistently correlated with abnormal semen parameters, increased DNA fragmentation, and poor ART outcomes (133, 134). Importantly, ORP can discriminate between fertile and infertile men with higher sensitivity and specificity compared with TAC or MDA (131). One of the key advantages of ORP lies in its reproducibility and clinical applicability. The assay requires minimal sample preparation, provides results within minutes, and demonstrates low inter-operator variability (135). This contrasts with conventional assays, which often require sophisticated equipment and lengthy protocols. Moreover, ORP integrates contributions from both oxidative insults (e.g., ROS, lipid peroxidation products) and antioxidant defenses, providing a holistic view of seminal redox homeostasis. However, ORP is not without limitations. While it offers an integrative measure, it does not identify specific sources of OS or pinpoint which antioxidant systems are deficient. Thus, ORP is more suited as a screening or monitoring tool rather than a definitive diagnostic assay (135). Furthermore, thresholds for defining pathological ORP remain under debate, with variability reported across populations and laboratories. This raises questions about generalizability and the need for region- or population-specific reference values.

Clinically, ORP holds significant promise for stratifying idiopathic infertility (136). Men with elevated ORP values but normal semen parameters may represent a ‘hyper-oxidative phenotype’ undetected by conventional diagnostics. Conversely, men with low ORP despite abnormal semen profiles may not benefit from antioxidant therapy, thereby avoiding the risk of RS. In ART settings, ORP could guide sperm selection or treatment strategies, improving embryo development and pregnancy outcomes (134). Thus, ORP represents a significant advancement in OS assessment, offering an integrative, rapid, and clinically relevant biomarker. While not a panacea, its adoption in diagnostic algorithms could reduce the reliance on the idiopathic label and enable mechanistically informed management of male infertility.

### 5.3 Emerging biomarkers: advanced oxidation protein products, 8-OHdG, and isoprostanes

Beyond conventional assays and ORP, a new generation of OS biomarkers has been investigated to capture specific molecular pathways relevant to sperm dysfunction. Among these, AOPP, 8-hydroxy-2′-deoxyguanosine (8-OHdG), and isoprostanes have shown particular promise, reflecting protein, DNA, and lipid oxidative damage, respectively (107, 137). AOPP are dityrosine-containing crosslinked protein products formed during oxidative modification of plasma proteins, particularly albumin, by chlorinated oxidants (138). Elevated seminal AOPP levels have been associated with impaired sperm motility, reduced fertilization rates, and increased apoptosis (137, 139). Unlike conventional protein carbonyl assays, AOPP measurement provides specificity for myeloperoxidase-mediated OS (140), often linked to leukocytospermia and inflammation (137, 141). This makes AOPP a valuable biomarker for identifying the inflammatory-oxidative endophenotype of idiopathic infertility. 8-OHdG is the most extensively studied biomarker of oxidative DNA damage. Its presence in sperm DNA or seminal plasma reflects guanine base oxidation, a lesion strongly associated with mutagenesis and compromised embryonic development (107). Elevated 8-OHdG levels correlate with poor ART outcomes, including recurrent implantation failure and miscarriage (142). Detection typically involves ELISA, immunohistochemistry, or HPLC with electrochemical detection, though standardization across platforms remains a challenge (107). Importantly, 8-OHdG provides mechanistic insight into the DNA-damage-dominant redox phenotype in idiopathic infertility.

Isoprostanes are prostaglandin-like compounds generated by non-enzymatic peroxidation of arachidonic acid, considered gold-standard biomarkers of lipid peroxidation (143). Their detection in seminal plasma or sperm membranes provides a stable, specific marker of oxidative lipid injury. Isoprostanes are less susceptible to artifactual generation during sample handling compared with MDA, enhancing their reliability (144). Elevated seminal isoprostane levels are consistently observed in infertile men (145) with poor motility and reduced fertilization competence (146), underscoring their role in the lipid peroxidation phenotype. Therefore, these emerging biomarkers offer molecular specificity, enabling the stratification of patients based on the predominant oxidative damage pathway. However, their integration into clinical practice faces obstacles. Assay cost, technical complexity, and lack of universally accepted thresholds limit widespread adoption. Furthermore, most studies remain observational, and prospective trials are needed to validate predictive value for ART outcomes. Nevertheless, the potential of AOPP, 8-OHdG, and isoprostanes lies in their ability to define mechanistic endophenotypes, protein oxidation, DNA damage, and lipid peroxidation, within the idiopathic category. Their use could enable precision diagnostics and guide targeted therapies, moving andrology closer to a mechanistically informed clinical framework.

### 5.4 Limitations of current methodologies: standardization, reproducibility, and thresholds

Despite advances in OS biomarkers, significant methodological limitations hinder their clinical translation. Issues of standardization, reproducibility, and threshold determination remain central obstacles, perpetuating the reliance on the idiopathic infertility label. Standardization is a major challenge. Different laboratories use diverse protocols for sample preparation, storage, and assay execution, leading to significant variability in reported values. For example, MDA measurements differ substantially depending on whether spectrophotometry, HPLC, or ELISA is used (147–149). Similarly, DNA fragmentation assays (e.g., TUNEL, Comet, SCSA) yield non-comparable results due to protocol heterogeneity (22, 107). This lack of standardization undermines biomarker reproducibility and limits cross-study comparisons, impeding the development of universal diagnostic criteria.

Reproducibility further complicates interpretation. Many OS assays are sensitive to pre-analytical variables such as abstinence duration, semen viscosity, and leukocyte contamination (150). Without rigorous quality control, results may reflect technical artefacts rather than true biological variation. Inter-operator variability and equipment calibration also contribute to inconsistent outcomes. These factors explain why conventional oxidative assays remain predominantly research tools rather than routine clinical tests. Threshold determination is equally problematic. While elevated levels of oxidative markers are consistently reported in infertile men, the precise cutoffs distinguishing fertile from infertile populations remain unclear. Variability in reference ranges across populations, combined with the influence of diet, lifestyle, and environmental exposures, complicates the establishment of thresholds. For instance, ORP cutoffs proposed in one cohort may not be universally applicable, thereby reducing clinical utility. Without validated thresholds, clinicians cannot confidently use oxidative biomarkers to guide therapy or prognosticate outcomes (151). Another limitation lies in the static nature of most assays. OS is a dynamic process influenced by episodic factors such as infection, fever, or lifestyle changes. Single-point measurements may not reflect long-term redox status (152), leading to misclassification. Longitudinal monitoring could improve accuracy but increase logistical and financial burdens. Clinically, these methodological limitations translate into diagnostic ambiguity. Patients with elevated biomarkers may not receive targeted interventions due to uncertainty about clinical significance, while others with subtle oxidative imbalances remain undetected. This diagnostic gap sustains the idiopathic category, where men are treated empirically with antioxidants or advanced ART without mechanistic stratification.

Addressing these limitations requires multi-centre standardization of protocols, establishment of population-specific thresholds, and validation of biomarkers in large, prospective cohorts. The integration of multiplexed assays or global measures, such as ORP, may mitigate variability; however, they too require rigorous validation. Ultimately, overcoming these methodological barriers is essential for embedding OS assessment into clinical andrology and reducing reliance on the idiopathic label.

## 6 Toward redox endophenotypes in idiopathic male infertility

### 6.1 Conceptualizing ‘endophenotypes’ in reproductive medicine

The term ‘endophenotype’ originates from psychiatric and neurobiological sciences (153, 154), where it was introduced to bridge the explanatory gap between observable clinical phenotypes and complex genotypic determinants. Endophenotypes are defined as measurable, heritable biological markers that lie closer to the underlying pathophysiology than clinical syndromes, thereby offering mechanistic insight into disease processes (33). In reproductive medicine, the application of endophenotyping remains nascent, but it provides a transformative framework for dissecting the heterogeneity of infertility, particularly IMI. In male reproduction, the conventional phenotype is typically expressed through semen parameters, sperm concentration, motility, and morphology. While these descriptors provide surface-level characterization, they fail to capture the molecular and cellular underpinnings of sperm dysfunction (155). Genetic testing, on the other hand, identifies specific chromosomal or Y-chromosome abnormalities but accounts for only a small fraction of infertility cases (20). Between these endpoints lies a significant gap, representing the molecular and biochemical processes that shape sperm function, an ideal terrain for endophenotyping. The rationale for conceptualizing endophenotypes in male infertility is threefold. First, it enables stratification of idiopathic cases into mechanistically defined subgroups, reducing reliance on exclusionary diagnoses. Second, it facilitates biomarker discovery by linking measurable intermediate traits, such as oxidative DNA damage or mitochondrial dysfunction, with reproductive outcomes. Third, it provides a framework for personalized interventions, allowing therapies to be tailored according to the dominant biological defect rather than empirical categorization.

Redox biology is particularly suited to endophenotyping because OS represents a convergent pathway integrating genetic predispositions, environmental exposures, lifestyle factors, and systemic metabolic states (156). Distinct oxidative profiles, ranging from lipid peroxidation to epigenetic instability, can be conceptualized as endophenotypes, each linked to specific mechanistic failures in sperm function. Importantly, these traits are quantifiable through biochemical, molecular, and functional assays, making them clinically actionable. Critically, endophenotypes are not static but dynamic, reflecting the interplay between intrinsic susceptibility and external exposures. This dynamism underscores their clinical value, as they can guide both diagnostic refinement and therapeutic monitoring. By embedding endophenotyping into reproductive medicine, idiopathic infertility can be redefined not as an absence of explanation but as a spectrum of redox-driven mechanistic subtypes. This paradigm shift is essential for achieving precision and improving ART outcomes.

### 6.2 Proposed redox endophenotypes

**Figure 2:**
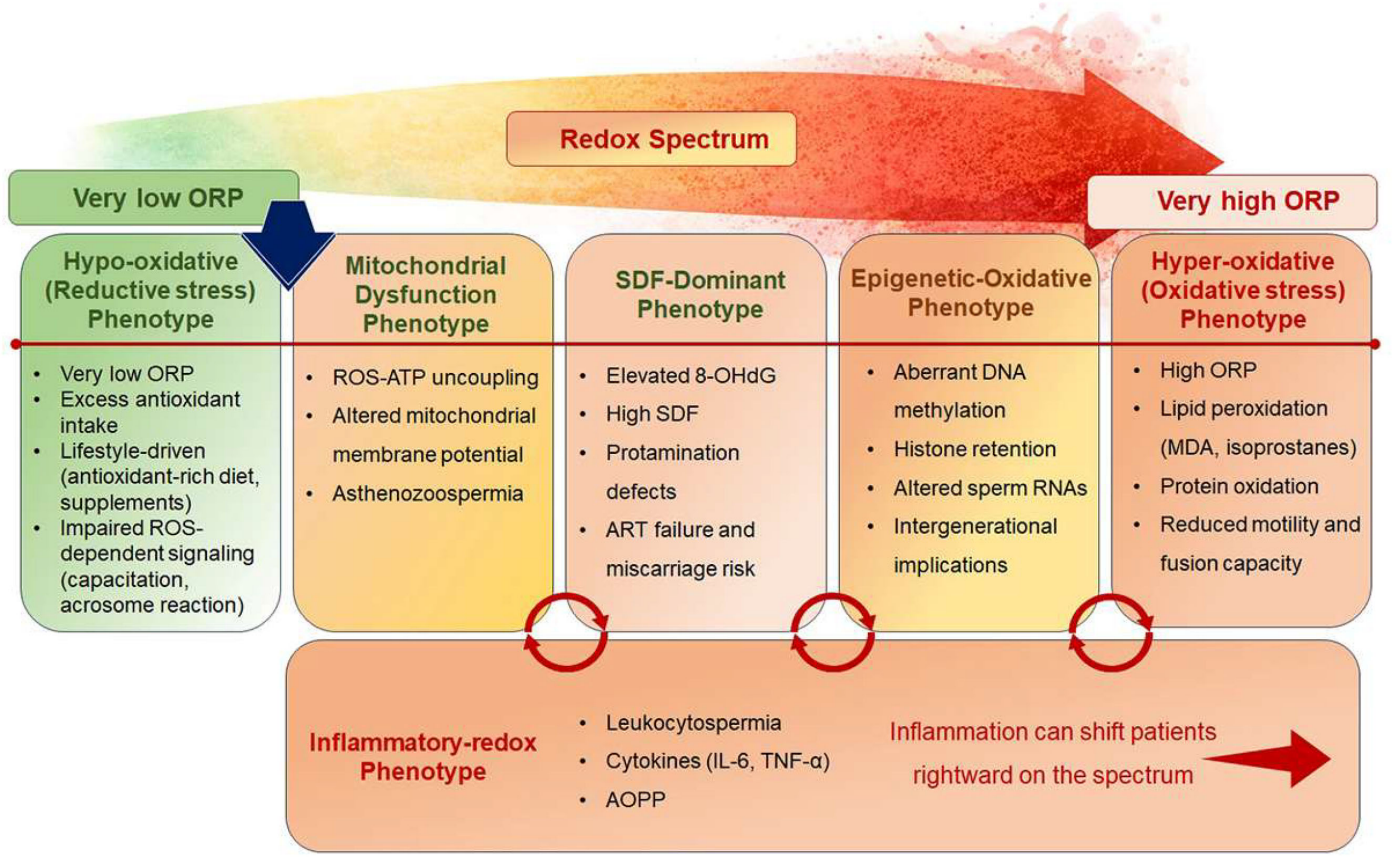
The redox spectrum of idiopathic male infertility illustrating redox endophenotypes across oxidative balance. Redox endophenotypes of IMI arranged along a continuum of ORP, ranging from very low ORP (hypo-oxidative/reductive stress) to very high ORP (hyper-oxidative stress). Each intermediate phenotype is associated with distinct molecular and functional abnormalities. An inflammatory-redox phenotype acts as an amplifier, promoting rightward shifts toward OS.

Redox endophenotyping proposes stratifying IMI into distinct mechanistic subgroups based on dominant oxidative pathways. Five major phenotypes have been conceptualized: hyper-oxidative, DNA damage-dominant, mitochondrial dysfunction, epigenetic-oxidative, and inflammatory-oxidative (**Table 2**). Each represents a quantifiable intermediate trait that links molecular dysfunction to clinical presentation. These five endophenotypes provide a structured framework for dissecting idiopathic infertility into mechanistically defined categories (**Figure 2**).

**Table 2:** Proposed eedox endophenotypes in idiopathic male infertility: biomarkers, clinical features, and ART implications.

Redox endophenotype	Key biomarkers	Mechanistic features	Clinical presentation	Implications for ART
Hyper-oxidative phenotype	Elevated ORP, MDA, Isoprostanes	Excess ROS, lipid peroxidation of sperm membranes	Reduced motility, poor fusion capacity	Antioxidant-enriched sperm preparation; avoid indiscriminate antioxidants to prevent reductive stress
DNA damage-dominant phenotype	Elevated 8-OHdG, High DFI, TUNEL/Comet positive	Oxidative DNA breaks, protamine deficiency	Normal semen parameters but recurrent ART failures, miscarriage	Advanced sperm selection (microfluidics, MACS); DNA integrity assessment before ART
Mitochondrial dysfunction phenotype	Reduced MMP, ATP, Oxidized mitochondrial proteins	ROS-ATP imbalance, impaired oxidative phosphorylation	Asthenozoospermia despite normal counts	Use of mitochondrial-targeted antioxidants (MitoQ, SkQ1); refined sperm selection
Epigenetic–oxidative phenotype	Abnormal methylation (H19/IGF2), Higher histone retention, Dysregulated sperm RNAs	ROS-induced epigenetic reprogramming defects	Unexplained ART failure, poor embryo quality	Lifestyle/dietary interventions; epigenetic biomarker–guided counseling
Inflammatory-oxidative phenotype	Elevated AOPP, Cytokines (IL-6, TNF-α), Leukocytospermia,	Leukocyte ROS, cytokine-driven redox imbalance	Normozoospermic infertility with high DNA damage	Combine antibiotics/anti-inflammatory therapy with antioxidants

#### 6.2.1 Hyper-oxidative phenotype (excess ROS, lipid peroxidation)

The hyper-oxidative phenotype is characterized by excessive ROS production exceeding antioxidant defenses, leading to widespread lipid peroxidation of sperm membranes. Biomarkers include elevated MDA, isoprostanes, and high ORP values (146, 151). Clinically, this phenotype manifests reduced sperm motility, impaired membrane fusion, and diminished fertilization rates. Such patients often respond to antioxidant therapy, although care must be taken to avoid RS. Identifying this phenotype is crucial to avoid indiscriminate antioxidant use and instead target individuals with demonstrable oxidative excess.

#### 6.2.2 DNA damage-dominant phenotype (oxidative genotoxic stress)

This phenotype is characterised by high levels of oxidative DNA damage, as reflected by biomarkers such as 8-OHdG and an increased DNA fragmentation index (22, 157). Protamine deficiency and incomplete chromatin condensation exacerbate vulnerability. Clinically, these men may have normal semen parameters yet exhibit recurrent implantation failure or miscarriage following ART. Empirical antioxidant therapy has shown inconsistent benefits, suggesting that targeted approaches, such as sperm selection techniques like microfluidic sorting, may be more effective. This phenotype highlights the importance of incorporating DNA integrity testing into diagnostic algorithms for idiopathic infertility.

#### 6.2.3 Mitochondrial dysfunction phenotype (ROS-ATP imbalance)

Mitochondrial dysfunction represents another endophenotype, where the overproduction of ROS coincides with impaired ATP synthesis, leading to motility defects. Biomarkers include reduced mitochondrial membrane potential, abnormal oxygen consumption rates, and oxidized mitochondrial proteins (158). Clinically, these men often present with asthenozoospermia despite otherwise normal semen parameters. This phenotype highlights the vicious cycle of ROS-induced mitochondrial damage perpetuating further ROS release. Mitochondrial-targeted antioxidants (e.g., MitoQ, SkQ1) offer promising interventions (94), though clinical validation remains limited.

#### 6.2.4 Epigenetic-oxidative phenotype (ROS-driven epigenome alterations)

Here, OS disrupts sperm epigenetics, manifesting as abnormal DNA methylation, histone retention, and altered sperm RNA profiles. These defects compromise embryo development and may have transgenerational health implications (109, 115). Biomarkers include altered methylation patterns at imprinted loci, increased histone retention, and dysregulated sperm miRNAs (115). Clinically, men with this phenotype often face unexplained ART failures despite normal semen and DNA fragmentation results. This phenotype emphasizes the need for integrating epigenetic biomarkers into diagnostics and considering lifestyle or dietary interventions aimed at restoring epigenomic balance.

#### 6.2.5 Inflammatory-oxidative phenotype (ROS-inflammation synergy)

This phenotype arises from the interplay between subclinical inflammation or infection and OS (48). Activated leukocytes release high levels of ROS, while inflammatory cytokines perpetuate redox imbalance (53). Biomarkers include elevated seminal leukocyte counts, cytokines (IL-6, TNF-α), and AOPP (121, 137). Clinically, this phenotype may be misclassified as idiopathic due to normal semen parameters, yet patients present with high DNA fragmentation and poor ART outcomes. Anti-inflammatory therapies, alongside antioxidants, may offer benefits, but precise stratification is essential to avoid overtreatment (54).

### 6.3 Linking redox endophenotypes with clinical subgroups (oligozoospermia, asthenozoospermia, teratozoospermia, normozoospermic infertility)

Integrating redox endophenotypes into existing clinical subgroups of male infertility offers a powerful tool for refining diagnosis and guiding therapy. Conventional categories, oligozoospermia, asthenozoospermia, teratozoospermia, and normozoospermic infertility, are descriptive but lack mechanistic resolution. By overlaying redox profiles, these phenotypes can be dissected into biologically meaningful subtypes. In oligozoospermia, reduced sperm counts may arise from hyper-oxidative damage impairing spermatogenesis or from inflammatory-oxidative processes disrupting the testicular microenvironment (159). Distinguishing these mechanisms through redox biomarkers can inform whether antioxidant or anti-inflammatory interventions are most appropriate. Asthenozoospermia is strongly linked to mitochondrial dysfunction. Identifying the mitochondrial dysfunction phenotype in this subgroup highlights the role of ROS-ATP imbalance and supports the use of mitochondrial-targeted therapies (160). However, some asthenozoospermic men may instead fall into the hyper-oxidative phenotype, necessitating distinct therapeutic approaches (161). Teratozoospermia often reflects oxidative damage to sperm DNA and proteins, implicating both DNA damage-dominant and epigenetic-oxidative phenotypes (161). In these patients, strategies such as sperm DNA integrity testing and epigenetic profiling may guide clinical decision-making and improve ART outcomes.

Normozoospermic infertility represents the most challenging subgroup, as semen parameters appear normal (162). Redox endophenotyping is particularly valuable here, revealing hidden defects such as oxidative DNA damage or epigenetic alterations (163). For these men, conventional diagnostics fail, yet ART outcomes remain poor, highlighting the clinical importance of uncovering underlying oxidative phenotypes. Ultimately, linking redox endophenotypes with clinical subgroups transforms descriptive diagnoses into mechanistic entities. This integration facilitates personalized interventions, avoids the pitfalls of empirical therapy, and enhances prognostic accuracy in ART. By embedding redox profiling into clinical practice, idiopathic infertility can be reclassified into distinct endophenotypes aligned with clinical presentation, paving the way for precision andrology.

## 7 Translational and clinical implications

The conceptual framework of redox endophenotypes holds significant translational potential for redefining IMI in clinical practice. As discussed, conventional approaches, which rely heavily on semen parameters and limited genetic testing, often fail to reveal the mechanistic underpinnings of infertility, thereby perpetuating the ambiguous ‘idiopathic’ label. By stratifying patients into biologically defined subgroups, clinicians can refine diagnostics, improve prognostication, and personalize interventions. This reclassification provides an opportunity to tailor antioxidant therapy to specific redox imbalances, thereby overcoming the ‘antioxidant paradox’ in which indiscriminate supplementation may yield inconsistent results or exacerbate RS. Importantly, redox profiling also lends itself to integration with high-throughput omics, including proteomics, transcriptomics, and metabolomics, enabling multidimensional characterization of sperm dysfunction and the development of biomarker-driven therapeutic strategies.

In ART, endophenotyping has practical implications for sperm selection, preparation media, and embryo outcomes. Patients with DNA-damage-dominant phenotypes may benefit from advanced sperm selection techniques, such as microfluidics, while those with hyper-oxidative profiles may require antioxidant-enriched preparation protocols. Recognition of mitochondrial or epigenetic defects could guide the adoption of targeted interventions, minimizing the risk of transmitting compromised paternal genomes to offspring. Prognostic algorithms incorporating redox biomarkers can further support clinical decision-making, informing couples about their reproductive prospects and guiding the escalation of treatment. While challenges remain in standardization, validation, and threshold determination, the translational promise of redox endophenotypes lies in transforming idiopathic infertility from a diagnosis of exclusion into a mechanistically informed condition. Such a shift not only enhances precision andrology but also aligns reproductive medicine with the broader vision of personalized healthcare, ensuring improved fertility outcomes and safeguarding intergenerational health.

## 8 Future perspective

Advancing the concept of redox endophenotypes requires rigorous validation and translation into clinical practice. Large, multi-center studies are essential to establish reproducible evidence, define population-specific thresholds, and correlate oxidative biomarkers with ART outcomes. Equally critical is the standardization of redox assays across laboratories, ensuring consistency in sample handling, analytical protocols, and clinical interpretation, similar to the global harmonization achieved for semen analysis. Emerging tools, such as ORP, provide integrative measures but require refinement to achieve universal clinical adoption. Parallel advances in artificial intelligence and machine learning can facilitate the integration of multidimensional datasets, enabling pattern recognition across redox, omics, and exposome variables, and supporting predictive clinical algorithms. The vision of precision andrology lies in combining redox biology with genetic susceptibilities and environmental exposures, constructing personalized profiles to guide interventions. Importantly, redox-driven sperm epigenetic modifications raise critical opportunities for intergenerational research, as paternal oxidative imbalance may influence embryonic development and offspring health. Investing in such studies not only refines male infertility diagnostics but also extends implications to long-term public health. Collectively, these perspectives highlight the promise of redox endophenotyping in transforming idiopathic infertility from a diagnosis of exclusion into a mechanistically informed, precision-based discipline.

## 9 Conclusions

Idiopathic male infertility remains a persistent diagnostic challenge because conventional evaluations fail to identify underlying molecular dysfunctions in a substantial proportion of patients. This review synthesizes current evidence supporting redox imbalance as a unifying but heterogeneous mechanism in idiopathic infertility and proposes redox endophenotypes as a structured framework for mechanistic stratification. By delineating hyper-oxidative, DNA damage-dominant, mitochondrial dysfunction, epigenetic-oxidative, and inflammatory-oxidative phenotypes, we highlight how redox profiling can refine diagnosis, rationalize antioxidant and adjunctive therapies, and inform ART decision-making. Adoption of redox endophenotyping, alongside validated biomarkers and integrative approaches, offers a practical pathway to move beyond exclusion-based diagnoses toward precision andrology.

## References

[1] Agarwal A Mulgund A Hamada A Chyatte MR . A unique view on male infertility around the globe. Reprod Biol Endocrinol. (2015) 13:37. doi: 10.1186/s12958-015-0032-1, PMID: 25928197 PMC4424520

[2] Boeri L Kandil H Ramsay J . Idiopathic male infertility–what are we missing? Arab J Urol. (2025) 23:215–29. doi: 10.1080/20905998.2024.2381972, PMID: 40747475 PMC12308862

[3] Assidi M . Infertility in men: Advances towards a comprehensive and integrative strategy for precision theranostics. Cells. (2022) 11:1711. doi: 10.3390/cells11101711, PMID: 35626747 PMC9139678

[4] Mannucci A Argento FR Fini E Coccia ME Taddei N Becatti M . The impact of oxidative stress in male infertility. Front Mol Biosci. (2022) 8:799294. doi: 10.3389/fmolb.2021.799294, PMID: 35071326 PMC8766739

[5] Pavuluri H Bakhtiary Z Panner Selvam MK Hellstrom WJ . Oxidative stress-associated male infertility: current diagnostic and therapeutic approaches. Medicina. (2024) 60:1008. doi: 10.3390/medicina60061008, PMID: 38929625 PMC11205999

[6] Sengupta P Dutta S Irez T . Oxidants and antioxidants in male reproduction: Roles of oxidative and reductive stress. J Integr Sci Technol. (2024) 12:753–3. doi: 10.62110/sciencein.jist.2024.v12.753

[7] Aitken RJ Drevet JR Moazamian A Gharagozloo P . Male infertility and oxidative stress: a focus on the underlying mechanisms. Antioxidants. (2022) 11:306. doi: 10.3390/antiox11020306, PMID: 35204189 PMC8868102

[8] Sengupta P Dutta S Saleh R . Assessment of seminal oxidative stress. In: Human semen analysis: From the who manual to the clinical management of infertile men. Cham, Switzerland: Springer (2024). p. 247–65.

[9] Homa ST Vessey W Perez-Miranda A Riyait T Agarwal A . Reactive oxygen species (ros) in human semen: Determination of a reference range. J assisted Reprod Genet. (2015) 32:757–64. doi: 10.1007/s10815-015-0454-x, PMID: 25749739 PMC4429439

[10] Baskaran S Finelli R Agarwal A Henkel R . Diagnostic value of routine semen analysis in clinical andrology. Andrologia. (2021) 53:e13614. doi: 10.1111/and.13614, PMID: 32400107

[11] Kaltsas A Dimitriadis F Zachariou D Zikopoulos A Symeonidis EN Markou E . From diagnosis to treatment: Comprehensive care by reproductive urologists in assisted reproductive technology. Medicina. (2023) 59:1835. doi: 10.3390/medicina59101835, PMID: 37893553 PMC10608107

[12] Singh A Soni A Kumari C . Understanding male infertility: A comprehensive review of causes, etiology, epidemiology, and risk factors. Cuestiones Fisioterapia. (2025) 54:2249–61. doi: 10.48047/k5sgh449

[13] Schilit SL . Recent advances and future opportunities to diagnose male infertility. Curr sexual Health Rep. (2019) 11:331–41. doi: 10.1007/s11930-019-00225-8, PMID: 31853232 PMC6919557

[14] Bisht S Dada R . Oxidative stress: Major executioner in disease pathology, role in sperm DNA damage and preventive strategies. Front Biosci (Schol Ed). (2017) 9:420–47. doi: 10.2741/s495, PMID: 28410127

[15] World Health Organization . Who laboratory manual for the examination and processing of human semen. In: Who laboratory manual for the examination and processing of human semen Geneva, Switzerland: World Health Organization (WHO Press) (2021).

[16] Kathrins M Niederberger C . Diagnosis and treatment of infertility-related male hormonal dysfunction. Nat Rev Urol. (2016) 13:309–23. doi: 10.1038/nrurol.2016.62, PMID: 27091665

[17] Barratt CL Björndahl L De Jonge CJ Lamb DJ Osorio Martini F McLachlan R . The diagnosis of male infertility: An analysis of the evidence to support the development of global who guidance—challenges and future research opportunities. Hum Reprod Update. (2017) 23:660–80. doi: 10.1093/humupd/dmx021, PMID: 28981651 PMC5850791

[18] Concepción-Zavaleta M Ibarra JLP Ramos-Yataco A Coronado-Arroyo J Concepción-Urteaga L Roseboom PJ . Assessment of hormonal status in male infertility. An update. Diabetes Metab Syndrome: Clin Res Rev. (2022) 16:102447. doi: 10.1016/j.dsx.2022.102447, PMID: 35272174

[19] McGriff SC Lo EM Hotaling JM Pastuszak AW . Optimal endocrine evaluation and treatment of male infertility. Urologic Clinics. (2020) 47:139–46. doi: 10.1016/j.ucl.2019.12.002, PMID: 32272985

[20] Hotaling J Carrell D . Clinical genetic testing for male factor infertility: Current applications and future directions. Andrology. (2014) 2:339–50. doi: 10.1111/j.2047-2927.2014.00200.x, PMID: 24711280

[21] Flannigan R Schlegel PN . Genetic diagnostics of male infertility in clinical practice. Best Pract Res Clin Obstetrics Gynaecology. (2017) 44:26–37. doi: 10.1016/j.bpobgyn.2017.05.002, PMID: 28601348

[22] Mottola F Palmieri I Carannante M Barretta A Roychoudhury S Rocco L . Oxidative stress biomarkers in male infertility: established methodologies and future perspectives. Genes. (2024) 15:539. doi: 10.3390/genes15050539, PMID: 38790168 PMC11121722

[23] Christoforaki V Venetis C Goulis DG Zepiridis L Chatzimeletiou K Mitsoli A . The role of seminal oxidation reduction potential in male infertility. A systematic review and meta-analysis. Reprod BioMedicine Online. (2024) 52:104858. doi: 10.1016/j.fertnstert.2024.08.281, PMID: 41207812

[24] Wu W La J Schubach KM Lantsberg D Katz DJ . Psychological, social, and sexual challenges affecting men receiving male infertility treatment: A systematic review and implications for clinical care. Asian J andrology. (2023) 25:448–53. doi: 10.4103/aja202282, PMID: 36412462 PMC10411259

[25] Ghanem H Shamloul R . An evidence-based perspective to the medical treatment of male infertility: A short review. Urol Int. (2009) 82:125–9. doi: 10.1159/000200785, PMID: 19321995

[26] Dashti MA Alhamar AY Shawky H Bakhiet M . Effectiveness of combined empirical therapies and double iui procedures in treatment of male factor infertility. Andrology. (2013) 2:2167–0250.100011. doi: 10.4172/2167-0250.1000112, PMID: 39887974

[27] Tournaye H . Male factor infertility and art. Asian J andrology. (2011) 14:103. doi: 10.1038/aja.2011.65, PMID: 22179511 PMC3735146

[28] Sengupta P Dutta S Jegasothy R Slama P Cho C-L Roychoudhury S . ‘Intracytoplasmic sperm injection (icsi) paradox’and ‘andrological ignorance’: Ai in the era of fourth industrial revolution to navigate the blind spots. Reprod Biol Endocrinol. (2024) 22:22. doi: 10.1186/s12958-024-01193-y, PMID: 38350931 PMC10863146

[29] Gualtieri R Kalthur G Barbato V Longobardi S Di Rella F Adiga SK . Sperm oxidative stress during *in vitro* manipulation and its effects on sperm function and embryo development. Antioxidants. (2021) 10:1025. doi: 10.3390/antiox10071025, PMID: 34202126 PMC8300781

[30] Esteves SC Roque M Bedoschi G Haahr T Humaidan P . Intracytoplasmic sperm injection for male infertility and consequences for offspring. Nat Rev Urol. (2018) 15:535–62. doi: 10.1038/s41585-018-0051-8, PMID: 29967387

[31] Rumbold AR Sevoyan A Oswald TK Fernandez RC Davies MJ Moore VM . Impact of male factor infertility on offspring health and development. Fertility sterility. (2019) 111:1047–53. doi: 10.1016/j.fertnstert.2019.05.006, PMID: 31155114

[32] Al-Kandari AM Alenezi A . Cost burden of male infertility investigations and treatments: A survey study. Urol Ann. (2020) 12:314–8. doi: 10.4103/UA.UA_48_20, PMID: 33776325 PMC7992531

[33] Liu C Gershon ES . Endophenotype 2.0: Updated definitions and criteria for endophenotypes of psychiatric disorders, incorporating new technologies and findings. Trans Psychiatry. (2024) 14:502. doi: 10.1038/s41398-024-03195-1, PMID: 39719446 PMC11668880

[34] Te Pas MF Madsen O Calus MP Smits MA . The importance of endophenotypes to evaluate the relationship between genotype and external phenotype. Int J Mol Sci. (2017) 18:472. doi: 10.3390/ijms18020472, PMID: 28241430 PMC5344004

[35] Ferlin A Dipresa S Delbarba A Maffezzoni F Porcelli T Cappelli C . Contemporary genetics-based diagnostics of male infertility. Expert Rev Mol Diagnostics. (2019) 19:623–33. doi: 10.1080/14737159.2019.1633917, PMID: 31215260

[36] Aitken RJ Baker MA . The role of genetics and oxidative stress in the etiology of male infertility—a unifying hypothesis? Front Endocrinol. (2020) 11:581838. doi: 10.3389/fendo.2020.581838, PMID: 33101214 PMC7554587

[37] Roychoudhury S Saha MR Saha MM . Environmental toxicants and male reproductive toxicity: Oxidation-reduction potential as a new marker of oxidative stress in infertile men. In: Networking of mutagens in environmental toxicology. Cham, Switzerland: Springer (2019). p. 99–115.

[38] Leisegang K Dutta S . Do lifestyle practices impede male fertility? Andrologia. (2021) 53:e13595. doi: 10.1111/and.13595, PMID: 32330362

[39] Boguenet M Bouet P-E Spiers A Reynier P May-Panloup P . Mitochondria: Their role in spermatozoa and in male infertility. Hum Reprod Update. (2021) 27:697–719. doi: 10.1093/humupd/dmab001, PMID: 33555313

[40] de la Iglesia A Jodar M Oliva R Castillo J . Insights into the sperm chromatin and implications for male infertility from a protein perspective. WIREs Mech Dis. (2023) 15:e1588. doi: 10.1002/wsbm.1588, PMID: 36181449

[41] McSwiggin H O’doherty A . Epigenetic reprogramming during spermatogenesis and male factor infertility. Reproduction. (2018) 156:R9–R21. doi: 10.1530/REP-18-0009, PMID: 29717022

[42] Tirpak F Hamilton LE Schnabel RD Sutovsky P . Biomarker-based high-throughput sperm phenotyping: Andrology in the age of precision medicine and agriculture. Anim Reprod Sci. (2024) 271:107636. doi: 10.1016/j.anireprosci.2024.107636, PMID: 39522272

[43] Sarigiannis D Karakitsios S Anesti O Stem A Valvi D Sumner SC . Advancing translational exposomics: Bridging genome, exposome and personalized medicine. Hum Genomics. (2025) 19:48. doi: 10.1186/s40246-025-00761-6, PMID: 40307849 PMC12044731

[44] Mani S Lalani SR Pammi M . Genomics and multiomics in the age of precision medicine. Pediatr Res. (2025) 97:1399–1410. doi: 10.1038/s41390-025-04021-0, PMID: 40185865 PMC12106083

[45] Chianese R Pierantoni R . Mitochondrial reactive oxygen species (ros) production alters sperm quality. Antioxidants. (2021) 10:92. doi: 10.3390/antiox10010092, PMID: 33440836 PMC7827812

[46] Aitken RJ . Impact of oxidative stress on male and female germ cells: Implications for fertility. Reproduction. (2020) 159:R189–201. doi: 10.1530/REP-19-0452, PMID: 31846434

[47] Tsai SC Lu CC Lin CS Wang PS . Antisteroidogenic actions of hydrogen peroxide on rat leydig cells. J Cell Biochem. (2003) 90:1276–86. doi: 10.1002/jcb.10738, PMID: 14635199

[48] Dutta S Sengupta P Roychoudhury S Chakravarthi S Wang CW Slama P . Antioxidant paradox in male infertility:’A blind eye’on inflammation. Antioxidants. (2022) 11:167. doi: 10.3390/antiox11010167, PMID: 35052671 PMC8772926

[49] Das S Roychoudhury S Roychoudhury S Agarwal A Henkel R . Role of infection and leukocytes in male infertility. In: Oxidative Stress and Toxicity in Reproductive Biology and Medicine: A Comprehensive Update on Male Infertility, vol. 1. Springer International Publishing, Cham (2022). p. 115–40. 10.1007/978-3-030-89340-8_6 35641868

[50] Chen H Alves MBR Belleannée C . Contribution of epididymal epithelial cell functions to sperm epigenetic changes and the health of progeny. Hum Reprod Update. (2022) 28:51–66. doi: 10.1093/humupd/dmab029, PMID: 34618012

[51] Chakraborty S Roychoudhury S . Pathological roles of reactive oxygen species in male reproduction. In: Oxidative stress and toxicity in reproductive biology and medicine: A comprehensive update on male infertility-volume one, vol. pp. Springer (2022). p. 41–62. 10.1007/978-3-030-89340-8_3 35641865

[52] Gil-Guzman E Ollero M Lopez MC Sharma R Alvarez J Thomas A Jr. . Differential production of reactive oxygen species by subsets of human spermatozoa at different stages of maturation. Hum Reprod. (2001) 16:1922–30. doi: 10.1093/humrep/16.9.1922, PMID: 11527899

[53] Henkel R Bastiaan H Schüller S Hoppe I Starker W Menkveld R . Leucocytes and intrinsic ros production may be factors compromising sperm chromatin condensation status. Andrologia. (2010) 42:69–75. doi: 10.1111/j.1439-0272.2009.00967.x, PMID: 20384795

[54] Izuka E Menuba I Sengupta P Dutta S Nwagha U . Antioxidants, anti-inflammatory drugs and antibiotics in the treatment of reproductive tract infections and their association with male infertility. Chem Biol Lett. (2020) 7:156–65.

[55] Joó JG Sulyok E Bódis J Kornya L . Disrupted balance of the oxidant–antioxidant system in the pathophysiology of female reproduction: oxidative stress and adverse pregnancy outcomes. Curr Issues Mol Biol. (2023) 45:8091–111. doi: 10.3390/cimb45100511, PMID: 37886954 PMC10605220

[56] Baszyński J Kamiński P Bogdzińska M Mroczkowski S Szymański M Wasilow K . Enzymatic antioxidant defense and polymorphic changes in male infertility. Antioxidants. (2022) 11:817. doi: 10.3390/antiox11050817, PMID: 35624681 PMC9138092

[57] Drevet JR . The antioxidant glutathione peroxidase family and spermatozoa: A complex story. Mol Cell Endocrinol. (2006) 250:70–9. doi: 10.1016/j.mce.2005.12.027, PMID: 16427183

[58] Micheli L Cerretani D Collodel G Menchiari A Moltoni L Fiaschi A . Evaluation of enzymatic and non-enzymatic antioxidants in seminal plasma of men with genitourinary infections, varicocele and idiopathic infertility. Andrology. (2016) 4:456–64. doi: 10.1111/andr.12181, PMID: 27027567

[59] Georgiou-Siafis SK Tsiftsoglou AS . The key role of gsh in keeping the redox balance in mammalian cells: Mechanisms and significance of gsh in detoxification via formation of conjugates. Antioxidants. (2023) 12:1953. doi: 10.3390/antiox12111953, PMID: 38001806 PMC10669396

[60] Bello T Ayo J Oyelowo B Khumran A Hassan R Oke-Ebgodo B . Antioxidative roles of ascorbic acid and tocopherol in semen preservation—a review. J Anim production Res. (2019) 31:65–72.

[61] Alahmar AT . The impact of two doses of coenzyme Q10 on semen parameters and antioxidant status in men with idiopathic oligoasthenoteratozoospermia. Clin Exp Reprod Med. (2019) 46:112. doi: 10.5653/cerm.2019.00136, PMID: 31408928 PMC6736512

[62] Henkel R Bittner J Weber R Hüther F Miska W . Relevance of zinc in human sperm flagella and its relation to motility. Fertility sterility. (1999) 71:1138–43. doi: 10.1016/S0015-0282(99)00141-7, PMID: 10360924

[63] Alahmar AT Sengupta P . Impact of coenzyme q10 and selenium on seminal fluid parameters and antioxidant status in men with idiopathic infertility. Biol Trace element Res. (2021) 199:1246–52. doi: 10.1007/s12011-020-02251-3, PMID: 32572802

[64] Kowalczyk A . The role of the natural antioxidant mechanism in sperm cells. Reprod Sci. (2022) 29:1387–94. doi: 10.1007/s43032-021-00795-w, PMID: 34845666 PMC9005387

[65] Cheng JW Ko EY . Causes of reductive stress in male reproduction. In: Oxidants, antioxidants and impact of the oxidative status in male reproduction. London, United Kingdom: Academic Press (2019). p. 55–64.

[66] Selvam MKP Agarwal A Henkel R Finelli R Robert KA Iovine C . The effect of oxidative and reductive stress on semen parameters and functions of physiologically normal human spermatozoa. Free Radical Biol Med. (2020) 152:375–85. doi: 10.1016/j.freeradbiomed.2020.03.008, PMID: 32165282

[67] Birk R . Nutrigenetics of antioxidant enzymes and micronutrient needs in the context of viral infections. Nutr Res Rev. (2021) 34:174–84. doi: 10.1017/S0954422420000244, PMID: 33081856

[68] Tvrdá E Massanyi P Lukáč N . Physiological and pathological roles of free radicals in male reproduction. In: Spermatozoa-facts and perspectives. London, United Kingdom: IntechOpen (2017).

[69] Zhang J Wang X Vikash V Ye Q Wu D Liu Y . Ros and ros-mediated cellular signaling. Oxid Med Cell Longevity. (2016) 2016:4350965. doi: 10.1155/2016/4350965, PMID: 26998193 PMC4779832

[70] Serafini S O’Flaherty C . Redox regulation to modulate phosphorylation events in human spermatozoa. Antioxidants Redox Signaling. (2022) 37:437–50. doi: 10.1089/ars.2021.0117, PMID: 34714121

[71] Thompson A Agarwal A Du Plessis SS . Physiological role of reactive oxygen species in sperm function: A review. In: Antioxidants in male infertility: a guide for clinicians and researchers. Springer Science and Business Media, New York, USA (2013). p. 69–89.

[72] Rivlin J Mendel J Rubinstein S Etkovitz N Breitbart H . Role of hydrogen peroxide in sperm capacitation and acrosome reaction. Biol Reprod. (2004) 70:518–22. doi: 10.1095/biolreprod.103.020487, PMID: 14561655

[73] Brener E Rubinstein S Cohen G Shternall K Rivlin J Breitbart H . Remodeling of the actin cytoskeleton during mammalian sperm capacitation and acrosome reaction. Biol Reprod. (2003) 68:837–45. doi: 10.1095/biolreprod.102.009233, PMID: 12604633

[74] Du Plessis SS Agarwal A Halabi J Tvrda E . Contemporary evidence on the physiological role of reactive oxygen species in human sperm function. J assisted Reprod Genet. (2015) 32:509–20. doi: 10.1007/s10815-014-0425-7, PMID: 25646893 PMC4380893

[75] Ahelik A Mändar R Korrovits P Karits P Talving E Rosenstein K . Systemic oxidative stress could predict assisted reproductive technique outcome. J Assisted Reprod Genet. (2015) 32:699–704. doi: 10.1007/s10815-015-0466-6, PMID: 25813658 PMC4429443

[76] Henkel R . Antioxidant paradox. In: Biomarkers of oxidative stress: Clinical aspects of oxidative stress, vol. pp. Cham, Switzerland: Springer (2024). p. 349–60.

[77] Lenzi A Picardo M Gandini L Dondero F . Lipids of the sperm plasma membrane: From polyunsaturated fatty acids considered as markers of sperm function to possible scavenger therapy. Hum Reprod Update. (1996) 2:246–56. doi: 10.1093/humupd/2.3.246, PMID: 9079417

[78] Aitken RJ . Free radicals, lipid peroxidation and sperm function. Reproduction fertility Dev. (1995) 7:659–68. doi: 10.1071/RD9950659, PMID: 8711202

[79] Kodama H Kuribayashi Y Gagnon C . Effect of sperm lipid peroxidation on fertilization. J andrology. (1996) 17:151–7. doi: 10.1002/j.1939-4640.1996.tb01764.x, PMID: 8723439

[80] Aitken RJ Gibb Z Mitchell LA Lambourne SR Connaughton HS De Iuliis GN . Sperm motility is lost *in vitro* as a consequence of mitochondrial free radical production and the generation of electrophilic aldehydes but can be significantly rescued by the presence of nucleophilic thiols. Biol Reprod. (2012) 110:111–1. doi: 10.1095/biolreprod.112.102020, PMID: 22933515

[81] Yeni E Ciftci H Savaş M Verit A Taşkin A . Is oxidative stress an etiologic factor in idiopathic male infertility? Turkish J Med Sci. (2010) 40:1–6. doi: 10.3906/sag-0907-6, PMID: 31411186

[82] Abruzzese GA Sanchez-Rodriguez A Roldan ER . Sperm metabolism. Mol Reprod Dev. (2024) 91:e23772. doi: 10.1002/mrd.23772, PMID: 39407445

[83] Pannu S Kumar P Baithalu R Pandey M . An overview of lipids, lipid peroxidation and antioxidants, and their impact on sperm. Indian J Anim Health. (2022) 61:235–41. doi: 10.36062/ijah.2022.04422

[84] Ribas-Maynou J Yeste M Salas-Huetos A . The relationship between sperm oxidative stress alterations and ivf/icsi outcomes: A systematic review from nonhuman mammals. Biology. (2020) 9:178. doi: 10.3390/biology9070178, PMID: 32708086 PMC7408105

[85] Kumar A Prasad J Srivastava N Ghosh S . Strategies to minimize various stress-related freeze–thaw damages during conventional cryopreservation of mammalian spermatozoa. Biopreservation biobanking. (2019) 17:603–12. doi: 10.1089/bio.2019.0037, PMID: 31429586

[86] O’Flaherty C Matsushita-Fournier D . Reactive oxygen species and protein modifications in spermatozoa. Biol Reprod. (2017) 97:577–85. doi: 10.1093/biolre/iox104, PMID: 29025014

[87] Demasi M Augusto O Bechara EJ Bicev RN Cerqueira FM da Cunha FM . Oxidative modification of proteins: From damage to catalysis, signaling, and beyond. Antioxidants Redox Signaling. (2021) 35:1016–80. doi: 10.1089/ars.2020.8176, PMID: 33726509

[88] Ribeiro JC Nogueira-Ferreira R Amado F Alves MG Ferreira R Oliveira PF . Exploring the role of oxidative stress in sperm motility: A proteomic network approach. Antioxidants Redox Signaling. (2022) 37:501–20. doi: 10.1089/ars.2021.0241, PMID: 34847748

[89] Bartesaghi S Radi R . Fundamentals on the biochemistry of peroxynitrite and protein tyrosine nitration. Redox Biol. (2018) 14:618–25. doi: 10.1016/j.redox.2017.09.009, PMID: 29154193 PMC5694970

[90] Wang S-B Murray CI Chung HS Van Eyk JE . Redox regulation of mitochondrial atp synthase. Trends Cardiovasc Med. (2013) 23:14–8. doi: 10.1016/j.tcm.2012.08.005, PMID: 23312134 PMC3936247

[91] Piomboni P Focarelli R Stendardi A Ferramosca A Zara V . The role of mitochondria in energy production for human sperm motility. Int J andrology. (2012) 35:109–24. doi: 10.1111/j.1365-2605.2011.01218.x, PMID: 21950496

[92] Hirose M Honda A Fulka H Tamura-Nakano M Matoba S Tomishima T . Acrosin is essential for sperm penetration through the zona pellucida in hamsters. Proc Natl Acad Sci. (2020) 117:2513–8. doi: 10.1073/pnas.1917595117, PMID: 31964830 PMC7007544

[93] Escada-Rebelo S Cristo MI Ramalho-Santos J Amaral S . Mitochondria-targeted compounds to assess and improve human sperm function. Antioxidants Redox Signaling. (2022) 37:451–80. doi: 10.1089/ars.2021.0238, PMID: 34847742

[94] Tiwari S Mohanty T Bhakat M Kumar N Baithalu R Nath S . Comparative evidence support better antioxidant efficacy of mitochondrial-targeted (mitoquinone) than cytosolic (resveratrol) antioxidant in improving *in-vitro* sperm functions of cryopreserved buffalo (bubalus bubalis) semen. Cryobiology. (2021) 101:125–34. doi: 10.1016/j.cryobiol.2021.04.007, PMID: 33933431

[95] Ribas-Maynou J Novo S Torres M Salas-Huetos A Rovira S Antich M . Sperm DNA integrity does play a crucial role for embryo development after icsi, notably when good-quality oocytes from young donors are used. Biol Res. (2022) 55:41. doi: 10.1186/s40659-022-00409-y, PMID: 36572948 PMC9791757

[96] Bao J Bedford MT . Epigenetic regulation of the histone-to-protamine transition during spermiogenesis. Reprod (Cambridge England). (2016) 151:R55. doi: 10.1530/REP-15-0562, PMID: 26850883 PMC4896072

[97] Drevet JR Hallak J Nasr-Esfahani M-H Aitken RJ . Reactive oxygen species and their consequences on the structure and function of mammalian spermatozoa. Antioxidants Redox Signaling. (2022) 37:481–500. doi: 10.1089/ars.2021.0235, PMID: 34913729

[98] Alvarez JG Gosalvez J . Role of protamine disulphide cross-linking in counteracting oxidative damage to DNA. In: Studies on men’s health and fertility. New York, NY, USA: Springer (2012). p. 221–35.

[99] Stavros S Potiris A Molopodi E Mavrogianni D Zikopoulos A Louis K . Sperm DNA fragmentation: Unraveling its imperative impact on male infertility based on recent evidence. Int J Mol Sci. (2024) 25:10167. doi: 10.3390/ijms251810167, PMID: 39337652 PMC11432134

[100] Kort DH Chia G Treff NR Tanaka AJ Xing T Vensand LB . Human embryos commonly form abnormal nuclei during development: A mechanism of DNA damage, embryonic aneuploidy, and developmental arrest. Hum Reprod. (2016) 31:312–23. doi: 10.1093/humrep/dev281, PMID: 26621855

[101] Musson R Gąsior Ł Bisogno S Ptak GE . DNA damage in preimplantation embryos and gametes: Specification, clinical relevance and repair strategies. Hum Reprod Update. (2022) 28:376–99. doi: 10.1093/humupd/dmab046, PMID: 35021196 PMC9071077

[102] Hammoud SS Nix DA Hammoud AO Gibson M Cairns BR Carrell DT . Genome-wide analysis identifies changes in histone retention and epigenetic modifications at developmental and imprinted gene loci in the sperm of infertile men. Hum Reprod. (2011) 26:2558–69. doi: 10.1093/humrep/der192, PMID: 21685136 PMC3157626

[103] Rashki Ghaleno L Alizadeh A Drevet JR Shahverdi A Valojerdi MR . Oxidation of sperm DNA and male infertility. Antioxidants. (2021) 10:97. doi: 10.3390/antiox10010097, PMID: 33445539 PMC7827380

[104] Borini A Tarozzi N Bizzaro D Bonu M Fava L Flamigni C . Sperm DNA fragmentation: Paternal effect on early post-implantation embryo development in art. Hum Reprod. (2006) 21:2876–81. doi: 10.1093/humrep/del251, PMID: 16793992

[105] McQueen DB Zhang J Robins JC . Sperm DNA fragmentation and recurrent pregnancy loss: A systematic review and meta-analysis. Fertility sterility. (2019) 112:54–60.e53. doi: 10.1016/j.fertnstert.2019.03.003, PMID: 31056315

[106] Dutta S Henkel R Agarwal A . Comparative analysis of tests used to assess sperm chromatin integrity and DNA fragmentation. Andrologia. (2021) 53:e13718. doi: 10.1111/and.13718, PMID: 32628294

[107] Agarwal A Farkouh AA Saleh R Hamoda TAAAM Salvio G Boitrelle F . Technical aspects and clinical limitations of sperm DNA fragmentation testing in male infertility: a global survey, current guidelines, and expert recommendations. World J men’s Health. (2023) 42:202. doi: 10.5534/wjmh.230076, PMID: 37635341 PMC10782128

[108] Kaltsas A Markou E Kyrgiafini M-A Zikopoulos A Symeonidis EN Dimitriadis F . Oxidative-stress-mediated epigenetic dysregulation in spermatogenesis: Implications for male infertility and offspring health. Genes. (2025) 16:93. doi: 10.3390/genes16010093, PMID: 39858640 PMC11765119

[109] Dutta S Sengupta P Mottola F Das S Hussain A Ashour A . Crosstalk between oxidative stress and epigenetics: Unveiling new biomarkers in human infertility. Cells. (2024) 13:1846. doi: 10.3390/cells13221846, PMID: 39594595 PMC11593296

[110] Lim DH Maher ER . DNA methylation: A form of epigenetic control of gene expression. Obstetrician Gynaecologist. (2010) 12:37–42. doi: 10.1576/toag.12.1.037.27556, PMID: 41147165

[111] Ito S Kuraoka I . Epigenetic modifications in DNA could mimic oxidative DNA damage: A double-edged sword. DNA Repair. (2015) 32:52–7. doi: 10.1016/j.dnarep.2015.04.013, PMID: 25956859

[112] Darbandi M Darbandi S Agarwal A Baskaran S Dutta S Sengupta P . Reactive oxygen species-induced alterations in h19-igf2 methylation patterns, seminal plasma metabolites, and semen quality. J assisted Reprod Genet. (2019) 36:241–53. doi: 10.1007/s10815-018-1350-y, PMID: 30382470 PMC6420547

[113] Park C-H Kim H-S Lee S-G Lee C-K . Methylation status of differentially methylated regions at igf2/h19 locus in porcine gametes and preimplantation embryos. Genomics. (2009) 93:179–86. doi: 10.1016/j.ygeno.2008.10.002, PMID: 18983907

[114] Miller D Brinkworth M Iles D . Paternal DNA packaging in spermatozoa: More than the sum of its parts? DNA, histones, protamines and epigenetics. Reproduction. (2010) 139:287–301. doi: 10.1530/REP-09-0281, PMID: 19759174

[115] Oliva R Ballescà JL . Altered histone retention and epigenetic modifications in the sperm of infertile men. Asian J andrology. (2011) 14:239. doi: 10.1038/aja.2011.159, PMID: 22057381 PMC3735090

[116] Santiago J Silva JV Howl J Santos MA Fardilha M . All you need to know about sperm rnas. Hum Reprod Update. (2022) 28:67–91. doi: 10.1093/humupd/dmab034, PMID: 34624094

[117] Conflitti AC Cicolani G Buonacquisto A Pallotti F Faja F Bianchini S . Sperm DNA fragmentation and sperm-borne mirnas: Molecular biomarkers of embryo development? Int J Mol Sci. (2023) 24:1007. doi: 10.3390/ijms24021007, PMID: 36674527 PMC9864861

[118] Leggio L Paternò G Cavallaro F Falcone M Vivarelli S Manna C . Sperm epigenetics and sperm rnas as drivers of male infertility: Truth or myth? Mol Cell Biochem. (2025) 480:659–82. doi: 10.1007/s11010-024-04962-w, PMID: 38717684 PMC11835981

[119] Fomichova O Oliveira PF Bernardino RL . Exploring the interplay between inflammation and male fertility. FEBS J. (2025) 292:3321–49. doi: 10.1111/febs.17366, PMID: 39702986

[120] Potiris A Moustakli E Trismpioti E Drakaki E Mavrogianni D Matsas A . From inflammation to infertility: how oxidative stress and infections disrupt male reproductive health. Metabolites. (2025) 15:267. doi: 10.3390/metabo15040267, PMID: 40278397 PMC12029481

[121] Irez T Bicer S Sahin E Dutta S Sengupta P . Cytokines and adipokines in the regulation of spermatogenesis and semen quality. Chem Biol Lett. (2020) 7:131–9.

[122] Liu K-S Mao X-D Pan F An RF . Effect and mechanisms of reproductive tract infection on oxidative stress parameters, sperm DNA fragmentation, and semen quality in infertile males. Reprod Biol Endocrinol. (2021) 19:97. doi: 10.1186/s12958-021-00781-6, PMID: 34183027 PMC8237428

[123] Henkel R . Infection in infertility. In: Male infertility: Contemporary clinical approaches, andrology, art and antioxidants. Springer (2020). p. 409–24.

[124] Arcaniolo D Favilla V Tiscione D Pisano F Bozzini G Creta M . Is there a place for nutritional supplements in the treatment of idiopathic male infertility? Archivio Italiano di Urol e Andrologia. (2014) 86:164–70. 10.4081/aiua.2014.3.164 25308577

[125] Grotto D Maria LS Valentini J Paniz C Schmitt G Garcia SC . Importance of the lipid peroxidation biomarkers and methodological aspects for malondialdehyde quantification. Quimica Nova. (2009) 32:169–74. doi: 10.1590/S0100-40422009000100032, PMID: 41711773

[126] Gupta S Finelli R Agarwal A Henkel R . Total antioxidant capacity—relevance, methods and clinical implications. Andrologia. (2021) 53:e13624. doi: 10.1111/and.13624, PMID: 32400041

[127] Seyedsadjadi N Berg J Bilgin AA Tung C Grant R . Significant relationships between a simple marker of redox balance and lifestyle behaviours; relevance to the framingham risk score. PloS One. (2017) 12:e0187713. doi: 10.1371/journal.pone.0187713, PMID: 29107974 PMC5673171

[128] Dorostghoal M Kazeminejad S Shahbazian N Pourmehdi M Jabbari A . Oxidative stress status and sperm DNA fragmentation in fertile and infertile men. Andrologia. (2017) 49:e12762. doi: 10.1111/and.12762, PMID: 28124476

[129] Nuhu F Gordon A Sturmey R Seymour A-M Bhandari S . Measurement of glutathione as a tool for oxidative stress studies by high performance liquid chromatography. Molecules. (2020) 25:4196. doi: 10.3390/molecules25184196, PMID: 32933160 PMC7571047

[130] Bartosz G . Superoxide dismutases and catalase. In: Reactions, processes: Oxidants and antioxidant defense systems. Berlin, Heidelberg, Germany: Springer (2004). p. 109–49.

[131] Agarwal A Henkel R Sharma R Tadros N Sabanegh E . Determination of seminal oxidation–reduction potential (orp) as an easy and cost-effective clinical marker of male infertility. Andrologia. (2018) 50:e12914. doi: 10.1111/and.12914, PMID: 29057493

[132] Agarwal A Sharma R Roychoudhury S Du Plessis S Sabanegh E . Mioxsys: A novel method of measuring oxidation reduction potential in semen and seminal plasma. Fertility sterility. (2016) 106:566–573.e510. doi: 10.1016/j.fertnstert.2016.05.013, PMID: 27260688

[133] Majzoub A Arafa M Mahdi M Agarwal A Al Said S Al-Emadi I . Oxidation–reduction potential and sperm DNA fragmentation, and their associations with sperm morphological anomalies amongst fertile and infertile men. Arab J Urol. (2018) 16:87–95. doi: 10.1016/j.aju.2017.11.014, PMID: 29713539 PMC5922185

[134] Panner Selvam M Henkel R Sharma R Agarwal A . Calibration of redox potential in sperm wash media and evaluation of oxidation–reduction potential values in various assisted reproductive technology culture media using mioxsys system. Andrology. (2018) 6:293–300. doi: 10.1111/andr.12461, PMID: 29314770

[135] Agarwal A Roychoudhury S Sharma R Gupta S Majzoub A Sabanegh E . Diagnostic application of oxidation-reduction potential assay for measurement of oxidative stress: Clinical utility in male factor infertility. Reprod biomedicine Online. (2017) 34:48–57. doi: 10.1016/j.rbmo.2016.10.008, PMID: 27839743

[136] Agarwal A Parekh N Selvam MKP Henkel R Shah R Homa ST . Male oxidative stress infertility (mosi): Proposed terminology and clinical practice guidelines for management of idiopathic male infertility. World J men’s Health. (2019) 37:296–312. doi: 10.5534/wjmh.190055, PMID: 31081299 PMC6704307

[137] Janiszewska E Kokot I Kmieciak A Gilowska I Faundez R Kratz EM . Are there associations between seminal plasma advanced oxidation protein products and selected redox-associated biochemical parameters in infertile male patients? A preliminary report. Cells. (2022) 11:3667. doi: 10.3390/cells11223667, PMID: 36429095 PMC9688436

[138] Piwowar A . Advanced oxidation protein products. Part i. Mechanism of the formation, characteristics and property. Polski merkuriusz lekarski: Organ Polskiego Towarzystwa Lekarskiego. (2010) 28:166–9. 20369749

[139] Kratz EM Piwowar A Zeman M Stebelová K Thalhammer T . Decreased melatonin levels and increased levels of advanced oxidation protein products in the seminal plasma are related to male infertility. Reproduction Fertility Dev. (2016) 28:507–15. doi: 10.1071/RD14165, PMID: 25218686

[140] Davies MJ . Myeloperoxidase-derived oxidation: Mechanisms of biological damage and its prevention. J Clin Biochem Nutr. (2010) 48:8–19. doi: 10.3164/jcbn.11-006FR, PMID: 21297906 PMC3022070

[141] Liu SX Hou FF Guo ZJ Nagai R Zhang WR Liu ZQ . Advanced oxidation protein products accelerate atherosclerosis through promoting oxidative stress and inflammation. Arteriosclerosis Thrombosis Vasc Biol. (2006) 26:1156–62. doi: 10.1161/01.ATV.0000214960.85469.68, PMID: 16497990

[142] Mukheef MA Ali RA Alheidery HHA . Follicular fluid 8-hydroxy-2-deoxyguanosine (8-ohdg) as biomarker for oxidative stress in intracytoplasmic sperm injection. J Med Invest. (2022) 69:112–6. doi: 10.2152/jmi.69.112, PMID: 35466131

[143] Basu S . Review isoprostanes: Novel bioactive products of lipid peroxidation. Free Radical Res. (2004) 38:105–22. doi: 10.1080/10715760310001646895, PMID: 15104204

[144] Fam SS Morrow JD . The isoprostanes: Unique products of arachidonic acid oxidation-a review. Curr medicinal Chem. (2003) 10:1723–40. doi: 10.2174/0929867033457115, PMID: 12871112

[145] Signorini C Moretti E Collodel G . Role of isoprostanes in human male infertility. Syst Biol Reprod Med. (2020) 66:291–9. doi: 10.1080/19396368.2020.1793032, PMID: 32842780

[146] Khosrowbeygi A Zarghami N . Levels of oxidative stress biomarkers in seminal plasma and their relationship with seminal parameters. BMC Clin Pathol. (2007) 7:6. doi: 10.1186/1472-6890-7-6, PMID: 17540046 PMC1906821

[147] Moselhy HF Reid RG Yousef S Boyle SP . A specific, accurate, and sensitive measure of total plasma malondialdehyde by hplc. J Lipid Res. (2013) 54:852–8. doi: 10.1194/jlr.D032698, PMID: 23264677 PMC3617959

[148] Bevan RJ Durand MF Hickenbotham PT Kitas GD Patel PR Podmore ID . Validation of a novel elisa for measurement of mda-ldl in human plasma. Free Radical Biol Med. (2003) 35:517–27. doi: 10.1016/S0891-5849(03)00359-9, PMID: 12927601

[149] Gomez Irvine Aitken . Evaluation of a spectrophotometric assay for the measurement of malondialdehyde and 4-hydroxyalkenals in human spermatozoa: Relationships with semen quality and sperm function. Int J Andrology. (1998) 21:81–94. doi: 10.1046/j.1365-2605.1998.00106.x, PMID: 9675617

[150] Khelfi A . Future directions, pitfalls, and solutions in oxidative stress assessment. In: Biomarkers of oxidative stress: Basics and measurement of oxidative stress, vol. pp. Cham, Switzerland: Springer (2024). p. 597–618.

[151] Agarwal A Roychoudhury S Bjugstad KB Cho C-L . Oxidation-reduction potential of semen: What is its role in the treatment of male infertility? Ther Adv Urol. (2016) 8:302–18. doi: 10.1177/1756287216652779, PMID: 27695529 PMC5004233

[152] Robert KA Sharma R Henkel R Agarwal A . An update on the techniques used to measure oxidative stress in seminal plasma. Andrologia. (2021) 53:e13726. doi: 10.1111/and.13726, PMID: 32814366

[153] Flint J Munafò MR . The endophenotype concept in psychiatric genetics. psychol Med. (2007) 37:163–80. doi: 10.1017/S0033291706008750, PMID: 16978446 PMC2829981

[154] Stoyanov DS . The endophenotype project and the validation theory: Integration of neurobiology and psychiatry. Folia Med. (2010) 52:18. 20380283

[155] Wang C Swerdloff RS . Limitations of semen analysis as a test of male fertility and anticipated needs from newer tests. Fertility sterility. (2014) 102:1502–7. doi: 10.1016/j.fertnstert.2014.10.021, PMID: 25458617 PMC4254491

[156] James SJ Melnyk S Jernigan S Cleves MA Halsted CH Wong DH . Metabolic endophenotype and related genotypes are associated with oxidative stress in children with autism. Am J Med Genet Part B: Neuropsychiatr Genet. (2006) 141:947–56. doi: 10.1002/ajmg.b.30366, PMID: 16917939 PMC2610366

[157] Hosen MB Islam MR Begum F Kabir Y Howlader MZH . Oxidative stress induced sperm DNA damage, a possible reason for male infertility. Iranian J Reprod Med. (2015) 13:525. PMC4637119 26568756

[158] Wang X Sharma RK Gupta A George V Thomas AJ Jr. Falcone T . Alterations in mitochondria membrane potential and oxidative stress in infertile men: A prospective observational study. Fertility sterility. (2003) 80:844–50. doi: 10.1016/S0015-0282(03)00983-X, PMID: 14505763

[159] Agarwal A Mulgund A Sharma R Sabanegh E . Mechanisms of oligozoospermia: An oxidative stress perspective. Syst Biol Reprod Med. (2014) 60:206–16. doi: 10.3109/19396368.2014.918675, PMID: 24815996

[160] Nowicka-Bauer K Lepczynski A Ozgo M Kamieniczna M Fraczek M Stanski L . Sperm mitochondrial dysfunction and oxidative stress as possible reasons for isolated asthenozoospermia. J Physiol Pharmacol. (2018) 69:403–417. doi: 10.26402/jpp.2018.3.05, PMID: 30149371

[161] Bdeir R Banihani SA . Role of pyridoxine and oxidative stress in asthenozoospermia. Heliyon. (2024) 10:e34799. doi: 10.1016/j.heliyon.2024.e34799, PMID: 39148988 PMC11325350

[162] Mayorga-Torres B Cardona-Maya W Cadavid A Camargo M . Evaluation of sperm functional parameters in normozoospermic infertile individuals. Actas Urológicas Españolas (English Edition). (2013) 37:221–7. doi: 10.1016/j.acuroe.2012.06.007, PMID: 23246107

[163] Venkatesh S Shamsi M Deka D Saxena V Kumar R Dada R . Clinical implications of oxidative stress & sperm DNA damage in normozoospermic infertile men. Indian J Med Res. (2011) 134:396–8. PMC3193724 21985826

